# Supplementary material for: Exploring user experience: A qualitative analysis of the use of a physical activity support app for people with heart failure
Source: PLoS One. 2025 May 22;20(5):e0309577. doi: 10.1371/journal.pone.0309577 (PMC12097600; doi:10.1371/journal.pone.0309577)
Supplement: S1 File — English_verbatim. (ZIP) [file pone.0309577.s001.zip › English_verbatim/ANLA032_eng.docx]

**ANLA032**

- You had heart failure, didn't you, that's right?

Yes, I've had heart failure for quite a long time, yes.

- Quite a long time, yes, and how long have you had this, you certainly have a screen like this at home?

Which screen are you thinking of?

- Yes, now I'm thinking about it ..

The screen I got from him, right?

- Yes?

He has been to pick it up.

- He's been there to pick it up, but you haven't had a screen before where you've weighed yourself and so on, have you?

Well, I have, but I was only supposed to have it for 6 months, but I've had it for 7 years now, I think.

- 7 months?

7 years.

- Oh, but he's picked that up now too, Andreas, hasn't he?

No.

- No, you still have it.

Yes, but I haven't used it in years.

- You don't have that.

I was only supposed to have it for 6 months.

- Well, okay, yes, there you see this other screen now that Andreas has just gone to pick up, that's what we're going to focus on a bit today in the interview, this activity coach part, this stick figure if you know what I mean?

Yes.

- Yes, that's great and I'll start with something general, what does physical activity mean to you?

It's when I've been out and walked something that I don't usually do, which is normal for me. I can only walk because I have osteoarthritis in one foot, so I can't do much else, I can't do anything. I have old handball injuries in one foot.

- Do you have it, oh, oh, oh.

So that I can't exert myself too much because then I get so damn sore.

- Yes, I understand.

And then I might have to remove my foot and get a prosthesis.

- Is that so?

If I get into too much trouble , yes.

- Oh, how boring.

So that's why you take it easy.

- I understand that, yes, because you're not that old.

No, apparently there are no good prosthetics for the feet.

- Oh well, we hope you can pull through then.

Hm so I'm holding back because if I go too much I'll get so damn sore that hm.

- You said something here at the beginning that I want to follow up on a little bit, you said something that you do out of the ordinary, can you elaborate on that a little more?

Yes, but I work at an HVB home so when I walk, I walk a little there but when I walk, when I'm outside and kind of walk more and it's not that common because I get pain. I can walk around 100 meters each time but if I feel like it's working a little then I walk a little further but I often have problems.

- So what you mean then, I'm thinking about this, when you say you do something else that's a little bit extra, is that what you're leaving?

Then I go for a normal little walk like normal people do.

- And that's roughly what you mean by physical activity for you?

Yes, yes, I can't go out and run, it doesn't work.

- Do you know any other physical activity, can you give an example of something else that you do that is physical activity for you?

I don't have time for that because I work full time, I'm a politician and I'm involved in the Swedish Hunters' Association .

- Dear someone .

.. so I don't have much time left for anything else.

- When you say the Hunters' Association, do you hunt?

Yes, but then I usually sit in maybe the towers very close to the road.

- Yes, okay.

I can't go out and walk in the forest because then I won't be able to move the next day.

- No, I understand, what is it for, is it possible that you are shooting, of course?

Yes, lots of wild boar and fallow deer.

- Yes, that's good, shoot the wild boar.

They have now decreased by 25%.

- Do they have?

Hmm.

- Well, I'll talk to you about that later, but we won't take it first. I'm wondering, you have, I told you, I know you have heart failure, and can you tell me how you think about physical activity in relation to having heart failure and whether you have any symptoms of heart failure?

Yes, I can have some days, I can have it so it feels a little heavy and breathing and stuff , I can do it. I almost think it's better when I don't take the medicine.

- You say?

Hmm.

- Yeah, okay, you're not doing that, are you?

Well, I do, I do that because I see that, what's it called, blood pressure and stuff like that, it gets better when I take the medicine.

- And you know, if you're thinking about quitting, don't do it because this is a long-term ...

No, no.

- .. yes, it's good for some people, and I understand that you don't want to take medication and so on, but maybe you can change it, I think, if it makes you feel bad?

I don't feel bad right away, but I feel like I get more breathless when I take the medicine.

- Yeah, but you have this thing about being out of breath, and if you're thinking about physical activity, can you relate to that and think a little about what it means to you?

You're too out of shape, that's how it is. I am, but I can't do anything because then I get so much pain that I can't even walk.

- No, so it's the foot, you mean?

Yes, you know, yes, that's what's stopping me right now. I've been an elite athlete...

- Yes, I understand.

.. so I've run a lot in my days so there's nothing that is, what's it called, that I don't want, would love to do, that is, move more.

- And this heart failure symptom that you say shortness of breath, is it something related to your activity or this, is it something that ...

Yes, it can be when I walk up stairs or I can feel it when I carry things, as soon as I have to exert myself a little more, I feel like I'm getting short of breath. I think it feels better when I don't take medicine...

- You can talk to your ...

.. I feel like I have more energy.

- Yes, I think you should talk to your doctor about that because it's not good. Because that feeling is not that fun.

No.

- But I wonder, what made you want to participate in this research project and did you have any expectations?

No, but when they call , I usually show up because I am on the Sörmland Healthcare Association.

- Yeah, how exciting.

Hm and then I know there's a problem.

- What did you say?

I know it's a problem getting a hold of people, so of course I'm volunteering.

- So this thing about wanting to participate is a bit grand for you, can you elaborate on that a bit?

No, but I think that as soon as they call and want there to be some kind of investigation and stuff like that , I'm happy to do it because I know that something good will come out of it.

- And you think there's good in that, do you want to develop it?

Yes, but it's often young people who call and it's something they do, that they're interested in or studying and researching, and if I can help them, I think it's good because it can help others in the future.

- So it's and helping others and it's young people who call you?

Yes, that's right, so far it's been younger people who have studied and done research and things like that.

- Did you have any expectations before you started participating in this study, about the activity coach itself, I'm thinking of now?

No, no big, no big expectations, I just wanted to help.

- Did you have any expectations because you say no big ones, you say, and then I wonder if you had any expectations?

No, but it's when you're involved in something like this, you realize, you become more clear-sighted that you're not moving enough, that's just the way it is.

- Yeah okay so that was you ..

That helps there ..

- It was a bit like you... well, sorry, what did you say?

It helped, you become blind when you just walk around and you just feel after it so that you don't get so much pain that you can't get up and walk, but if you can do a little more every day, something extra, it helps.

- Was that how you thought before you entered the study now or was it something you took with you when you had ...

Which I brought with me.

- So this means you became a little more clear-sighted when it comes to physical activity?

Yes, yes, you have to, it puts pressure on you, pushes you a little bit, at least, that's why I have to start thinking a little more about this and try as hard as I can.

- Exactly, can you tell us a little about your experiences using the activity coach?

Oh no, but it's not every day that you remember, it's important to have it in a place that you see it all the time, I feel, that you have it, it's in front of you, otherwise it's easy to forget and click on it that you've done some activity or something like that , so I think you should have that more in front of you. If you put it away or something like that, then it's easy to miss, forget and click on it.

- Yes, so that's what you noticed and you had to present it then in order to be able to ..

Yes, be visible at all times.

- Where was it with you then?

I had it in the kitchen on the sink, I put it out there.

- How, and how did you use that activity coach then?

I used to press it when I got home from work or something like that , but then sometimes I would do other things and then I would miss it when I got home.

- What did you do then?

Yes, it sometimes skipped to a new day.

- And could you go back, right?

No, I don't think so.

- No, exactly, has this activity coach influenced you?

Yes, actually positive. As I say, you've thought about how little activity I actually do, so you think about it in a different way, that you try, no, but I can walk for 10 minutes or so. You can go for a short walk that you didn't do before because you were kind of scared, but it has had a positive effect on me.

- It has had a positive impact on you, so you took an extra round because you had it at home, does that sound like it?

Yes, I have to take a 10- minute round just so I can push it, I thought.

- Yes, you thought so, yes?

Yes.

- And do you have it on... the activity coach, I hear that a little bit, but has it affected your physical activity?

Yes, it has actually done that. You actually think about going for a walk and I've noticed, I sit in meetings a lot, a lot and sometimes from one meeting to another for example. Before then, you went and sat down and had a cup of coffee, now you go out and just walk around the block maybe but I get hurt but I feel like it's getting better.

- And what is getting better?

No, but I feel like I'm getting more alert and I'm getting a little more oxygen and stuff like that actually.

- So, what is it called ...

The little that does help.

- How much more do you think you've started moving now that you've had this stick figure that I'm talking about?

If I take it like before these weeks, I'll move maybe one to two times more a day, yes, I do, yes.

- Exciting.

It's short, it's maybe 10 minutes or 5 minutes or something like that but I kind of have instead of sitting down right away so that, like I did before so as not to get hurt, I go out. It can just be a little round, can it be?

- Sorry, what did you say?

No, but it has become more positive, it has become just, you get a little more oxygenation and stuff .

- And what do you mean by oxygenation, can you describe it?

No, but I sit in meetings a lot and the air is bad and stuff . Now I go outside and stretch and move around a bit and it gets better. You get more alert.

- Feeling more energetic?

Yes, I feel it.

- Anything else you experienced physically or mentally or in any way?

No, nothing more than I miss moving around more.

- Yes and that's something that you've discovered by now, right ?

No, I've discovered that before too, but, more so now that I miss this life of mine, as an athlete before, that you could do a lot more before. You could play a little badminton with some guys. Today you can't do that, no, then you have to sit and watch.

- Yes, I understand that it's hard.

Yes, yes.

- Yes, that's perhaps what I was going to ask here, but did you have any negative experiences using this activity coach?

No, I think it was only positive.

- Because then this question comes ...

The only negative thing was maybe that you got a little, when you missed sometimes, just damn, you got a little negative.

- What was it, how did you experience it?

No, but it was just that if you missed it, you missed it and you pressed, damn I should have done that, I missed that yesterday. You got a little anxious about having stood up for yourself, but I'm a little special because I'm in the healthcare union and stuff like that too.

- What do you mean by that, you're a little, what do you mean by that?

You mean I'm a little special, no, but I'm passionate about it. I'm passionate about healthcare and stuff like that.

- But you, this anxiety, how did you experience it then, was it like ...

No, it's not a big anxiety, it's mostly that you, the freak, that you get disappointed in yourself and that you didn't push through because it was a bit of a shame that you couldn't go back actually. Like sometimes I'm at meetings maybe 200 miles away and stuff like that sometimes and then you come home. The meetings are until 10 o'clock in the morning .. 9-10 at night and then you come home and then at 12 at night it's over.

- Yes, exactly.

Then I couldn't push back like that.

- I understand.

Yes, I missed quite a few.

- How many times, how much did you use the activity coach?

I tried to use it every day and push.

- You tried, yes.

Yes, yes.

- And how was it and ..

You missed it then, I did because I was home late and stuff.

- Yes, I understand and I wonder, how did you experience, what was it like to register in this stick figure, what did you think, can you ..

It's easy, it's really easy so there were no surprises.

- No, no weirdness, right?

No.

- No, then it was a bit like this that you could, every week, the activity was summed up on the screen, have you looked at it?

Hmm.

- Could you set goals for the coming week?

Yes, but I haven't done that, I haven't done that.

- No, but have you looked at the summary of yours or do you have it because it happens automatically?

Yes, at some point I looked at the summary, I did, but you would want much, much more.

- What do you mean then?

We would have liked to have had much more activity.

- How did you experience it when you watched it then for ..

No, but it was, it was a wake-up call, I have to try, I have to try.

- Yes, it was an alarm clock and see that?

Yes, it becomes a wake-up call for one because one often gets into a rut. This as I said then, meeting means there is a little break yes then you sit down and then have a cup of coffee. Now I have kind of thought more like no but I go outside.

- Yes, that needs, I would also like to be part of a study sometimes because I also need to be reminded.

Yes.

- But you, it was also possible to look at something called history via a tab where you could, how you had registered activity in previous days and weeks, did you ever do that?

No, no, I didn't.

- You didn't do it but you knew it existed?

Yes, I saw that there was that tab, but I never went into it.

- No, and that wasn't something you were shown there at the beginning?

Well, he probably showed it at the beginning, I think he did, but yeah.

- Yes, because you looked, I know you can see something like this, you get the first week there were no goals but then next week, I think there will be some kind of red line and you should try then, but you didn't look at it?

No, I never looked at it.

- How come, was there something in particular that made you not watch it?

No, it wasn't anything special, no, it wasn't, no.

- Did you use ..

I thought I was fully enlightened that I was doing too little.

- Yes, I understand, yes, but you, did you use this activity coach in any other way than that...

No, I didn't.

- And then I wonder, I've already asked that, you used the activity coach almost every day and was it a lot, you think, or was it a little... or a lot or a little use, or how did you think it was, can you assess that?

It's a little, a little use. It doesn't take many seconds to do that, no.

- No, so it was of little use?

Yes, it's not a big burden as you can keep up with it.

- And was that roughly how you had envisioned using it?

Yes, the positive thing was that I hadn't thought about it, it was like discovering how little you move, that has been positive.

- You experience it as positive, yes.

Yes, yes.

- How do you think about the future now?

Going forward, I definitely plan to not sit down and have a cup of coffee like that, but I'm going to go out a lot more because I have, I sit on 12 different chairs in the municipality.

- Oh my God!

Plus the Hunters' Association then, which I'm also on the board of, so hm, and so I work full-time.

- Yes, I hear that and then you have time with me, that's good.

Yes, I'm going to the social services committee soon.

- Yes, oh, oh, I would like to talk to you more, we'll have to do that a little later here, but you..

Yes, yes, but it's okay, it's after lunch.

- Do you see anything that could be developed with this activity coach to make it better?

Well, I think the weight is pretty good when you weigh yourself. The last one that I had that I have here at home, which is here at home, I thought it was good that you weighed yourself because then you could see all the time, you became whole, you got up.. like upgraded all the time.

- But you did, couldn't you now in this screen?

No, it wasn't waves or anything like that now.

- It was just the activity coach in it, yes. But what about the weight, you would want that too, wouldn't you?

Yes, like I had last time, it was good. Then you stood on that screen and pressed the buttons at the same time.

- Yes, that and this particular activity, the stick figure, was there something that didn't work that wasn't good?

No, I think it gave me what I needed, I felt.

- It gave you what you needed.

Yes.

- And you may have said it before but is there anything else you want to add to this that you needed something?

No, but that thing about the importance of being able to stand up, that's what I actually missed because I've been there before.

- Yes, exactly, what would have made you want to use this stick figure more?

Oh... I don't know, uh, I don't know actually. It could have been that it had turned green when it was positive and red when it was negative somehow.

- Some kind of signal?

Yes.

- Would you have liked that?

So fast, yes, that it became green maybe if you were ever above average, if you say, average activity, it becomes green because then you want, then I think you want green. You work more to get green.

- Yes, exciting.

Yes, and if you are moving around a lot during that time, it might turn red that day.

- Yes, we will take that with us.

Yes, because then you kind of fight to try to get that green every single day.

- Yes, I get it, interesting. If you were offered to continue using this stick figure, the activity coach, would you, how would you view it?

Yes, I'm no stranger to that actually.

- Can you elaborate a little on why you feel that way?

No, because it has helped me and can it continue to help me. Now I kind of have to think about it so that I don't go back to the old ways.

- And you're talking about physical activity now?

Yes.

- Anything else?

Well, that's the thing about weight, because then you can kind of follow it in a completely different way than you do today.

- I wonder if you have anything else you would like to reflect on the study or highlight or tell me now before we conclude?

When I was there last time with the weight, it probably gave me more than this one did too. The scale was there.

- But then the activity coach wasn't there, was he ?

No, it wasn't.

- No, exactly that and how come it, what do you think about the weight, was it like ..

You should have a combination of both, so both the activity coach and the weight.

- Yes, that's right, you want both there, yes, and that's what you're thinking about, is it your heart failure diagnosis that you're thinking about or are you thinking that you ...

Yes, yes absolutely, hm.

- Yes, but you, anything else you want to add before we finish?

Hm, yes, if I were to get a scale, I would also include what's it called, pulse and stuff like that because it's really important because you can see a lot about how you're feeling there. Is it good today or not.

- So the pulse too?

Yes, both upper and lower pulse. I touch my arm sometimes ..

- When you say upper and lower, do you mean blood pressure?

Yes, yes exactly. I take it sometimes and I notice if I see that the pulse is high in the morning and then the other values are usually a little wrong too. You become more aware of it, as it were.

- Yes, so you would like to have both weight and pulse and blood pressure?

Yes, because if you get it in the same wave, just fuck it ..

- And does it glow green or red?

Yes, it would have been.

- take care of that, you know, I think.

Yes, that's good.

- But you, I'll turn off the recording here then, thank you.
