# Supplementary material for: Exploring user experience: A qualitative analysis of the use of a physical activity support app for people with heart failure
Source: PLoS One. 2025 May 22;20(5):e0309577. doi: 10.1371/journal.pone.0309577 (PMC12097600; doi:10.1371/journal.pone.0309577)
Supplement: S1 File — English_verbatim. (ZIP) [file pone.0309577.s001.zip › English_verbatim/BEJO106_eng.docx]

**BEJO106**

- First question here, let me just say what date it is today, August 28th, and then we'll see here, if you could say what physical activity means to you, what do you think when you hear it?

Well, maybe you should just go out and walk a bit.

- Can you think of any other examples of what could be physical activity for you?

I actually can't because my chest is so heavy and stuff like that, I can't bear it so much that I can walk for a walk, maybe a block or so. Then I can't bear it anymore.

- Exactly, I had a follow-up question there which was, tell me how you think about physical activity in relation to the fact that you have these symptoms. Now you may have already answered that, but does it affect you, so to speak?

Which one do you mean?

- These symptoms you talk about, like your chest feels heavy and you get short of breath and stuff?

Yes, it does if I walk too far, but I can walk certain distances, it's fine, but not, I can walk around the block where I live and then it stops.

- Hm, do you remember what made you want to participate in this research project? You agreed to be part of it, so to speak. How did that come about?

Yes. I did.

- Why did you accept, if I may ask, how did it come about?

Yeah, I don't know, I thought it was a good purpose.

- Yes, did you have any expectations for this project before you started?

No, I didn't, I know how..., I know how much I can handle anyway. I don't know if I had, that I would get better from this or not.

- No, if we're talking about the activity coach, as I called it then, that you could register how many minutes you were active, can you tell us a little about how you experienced it, your experience of using it?

Well, I don't know what to say about that. I'm going for a walk and it's not very long actually. I don't know what to say.

- But did you experience that the actual use of this activity coach to indicate how much you move affected your physical activity?

It affects my body, this breathing, that I can't bear anything, I can't bear to go too far or have activities that are too big for me, then it stops.

- Did you experience, yes forgive me?

Yes, then I'll be completely out of breath. This is difficult.

- Yes, I understand, I understand, did any of you experience any negatives or did you have any negative experiences from being part of this project and using this activity coach?

That, I can't say, but I did the best I could.

- Yes, you had it the other way around ..

Nothing negative.

- No.

No, I didn't think so, but I tried to do the best I could.

- Exactly, and did you have any positive experiences instead ?

Well, what can I say, that happened too because then I put in more effort than I do when I just sit still. Then I actually offered a little more, I have to admit.

- Okay, if we think a little more about this screen where you could register physical activity, I think it was your husband who helped you specifically with this, but what was your experience of being able to continuously register how much physical activity you had been on this screen?

Yes, I don't know what to answer that, it was difficult, it was a difficult question.

- Yes?

No, I can't answer that.

- Something that I'm not sure you necessarily noticed, but I'm asking the question anyway, after each week, you had this for 12 weeks during the spring and every week, it was probably on Monday then or Sunday at the weekly shift anyway, your activity was summed up on the screen and you could then set a goal for the coming week as well, was that something that you noted?

Yes, I don't remember that, I must say.

- No

No, it was very difficult, I don't remember much, you know.

- No, I understand, it was a while ago now, wasn't it?

Yes, that's right, so I don't have any, no, I don't remember much of that now.

- No, that's fine, that's no problem. Let's see what I had written then, that's it, then again this may be something that you may not remember but there was also an opportunity to look at how much had been registered then in previous days and previous weeks, did you ever look at that and sort of compare or in some other way look at how you registered?

No, I don't think I have. Because when I can do it, you can do it. I can't outdo myself.

- No, but it sounds like a healthy attitude anyway, I understand that.

I can't say that, no, those were difficult questions you brought up here.

- But that's how it is, man.... time flies and there's a whole summer in between too.

During that time, people have forgotten about it.

- I understand that, you're not the only one, I can say you 're in good company, but you know what, those little golden cards come out of your experiences anyway, so I'm grateful that you take your time just as much.

Yes, that's lucky, because I don't think I have the answers to most things.

- You don't have to think about that, as I said, I'm just grateful on behalf of the university, but I have a handful of more questions here that I thought I'd pester you with.

Yes

- Then we'll see here, again then connected to this then activity coach, did you use it in any other way than what we've talked about or did it arouse any other thoughts and associations or behaviors in you, would you say, or discussions or questions?

What do you mean, should I go out and walk or not or what?

- Yes exactly, I don't want to put words in your mouth here, but there's more to it, we talked about being able to record how much you've moved. You yourself mentioned that it made you think a little more about going out and walking, etc. so I guess I'm just going to spin it further and think about, were any new thoughts and considerations about physical activity born in the wake of this?

I try to think that I need to move more than I do. And try to go for a short walk, whatever I can manage. I think so, I have that feeling. I need to move, and I do more or less. Well, it wasn't easy to not answer this and make sense.

- We're moving forward, it's going well. Do you have any idea how much you used this activity coach and by that I mean how often you or with the help of your husband then recorded physical activity, how often you were in there fiddling with this, so to speak?

No, but I'm thinking about it a little more now maybe... .. that I need to move a little more. I'm fighting for it anyway, that now I have to go for a walk, or well... then I'll do it, move a little more than I might have done.

- Yes, but that's great. And if you think back to the actual experiment and that you could then on this screen, this tablet or tablet computer, whatever you want to call it, you could then say yes, but today I had moved for 20 minutes, for example , do you have any sense of how often you did that, that is, how many days in a week you did this?

No, we haven't noted that in any way, no. Because you're sometimes at home and sometimes at the summer cottage, we haven't thought about it at all right away.

- No, if we say it like this...

I must admit.

- Well ..

The days just pass here…

- If we say this instead .

won't get many answers from me today.

- Well, as we move forward, I'm very satisfied so far so don't think about it. But I understand that you didn't kind of keep a diary of how much you used, but it was more of a feeling there, but if I rephrase the question, as much as you used it, would you say that it was roughly in line with what you had in mind at the beginning of the experiment or did you do more or less, is that possible to answer?

It was pretty much as I had imagined, I think, and then it was easier to keep fighting in a different way than the days are passing anyway now, if you know what I mean.

- Yes absolutely, absolutely, that's great. So let's see here, if we look ahead or try to think a little critically maybe, was there something you were missing or something you thought about during this project, some feature, some functionality or something, man, something that you were simply missing with this activity coach that would have made it better?

No. I don't know, no, I don't feel that, no.

- Did you encounter any problems or issues with the operation?

No, I don't think so, but if I can handle it, I can handle it and then... ... so I don't think so, no.

- It wasn't something you...

I'm trying my best.

- Yes, exactly that, and there was nothing you were missing in the way you indicated how much you had moved and so on, there was nothing you were missing in the procedure, so to speak, that would have made it better or easier, right?

I don't know, it's hard to say.

- You mentioned yourself that you move around a bit between when you're at your summer home and in town and so on, but with the exception of that, is there anything that would have made you want to use it more often? And now I don't mean going out and walking more often without being more active in registering?

That's what happens. When I'm in town, I actually walk more, you're out in a different way, and when I live in an apartment, you don't go out and walk.

- Yes, exactly that, but if we exclude if we don't think about how much you actually walk or how much you actually move, now I'm more interested in registering this how much, going into the tablet itself and the screen and indicating how much you moved, that particular use, was there anything that would have made you want to use this, this system?

No , we didn't do that, it was very bad.

- And if you were offered the option to continue using the activity coach after the 12-week period, how would you feel about it?

No, I don't think I would want to.

- And can you elaborate on what makes you feel that way?

I don't think so, it doesn't matter what I have to offer. I can't stand anything, I mean, I'm just getting worse and worse and then I think it's almost unnecessary.

- Yes, it can feel gloomy, I can understand that.

Yes, it does.

- Then I really only have one more question and that is if you have anything else that you would like to reflect on, shed light on or send to me before I sit and think about how we should move forward with this research?

That's difficult, no, what could it be, no, I have nothing to say about that.

- Well, no, but then you hear me , that was the last question I had. I have to say thank you very much for calling and surprising me.

Good luck with the next one, if you get a better answer.

- I'm grateful anyway and I say thank you for the help and hope you're doing well in the rain, thank you very much.
