# Supplementary material for: Exploring user experience: A qualitative analysis of the use of a physical activity support app for people with heart failure
Source: PLoS One. 2025 May 22;20(5):e0309577. doi: 10.1371/journal.pone.0309577 (PMC12097600; doi:10.1371/journal.pone.0309577)
Supplement: S1 File — English_verbatim. (ZIP) [file pone.0309577.s001.zip › English_verbatim/BELU114_eng.docx]

**BELU114**

- Then I'm just thinking, just tell me a little bit, it's about this activity coach that you have in your, that you've had in this screen that you have at home, right?

Yes.

- And it's not this one, where you measure weight and adjust your medications, but this part, this stretcher, and then you understand what I'm saying, what I mean?

Yes, yes.

- How good. But then I wonder, the first question is just if you want to tell me what physical activity means, what it means to you?

Yes, it's simply that I can move, that you don't become sedentary.

- Yes and can you, can you give examples of physical activity, what that means and being able to move for you?

Yes, that you are outside and, for example, outside walking.

- Yes, going out for a walk could be an example?

Yes.

- Anything else that comes to mind when I say physical activity?

Yes, you can do your homework, clean and keep going.

- Yes, to cope with your job at home, you think?

Yes, exactly.

- How great, then I'm wondering, what do you think about physical activity in relation to having heart failure?

Yes.. it's how much you can move so that you, yes, how, it's how you can move, how much you can move simply, yes.

- And if you have, I know you have heart failure and from time to time you can have slightly different symptoms, what do you think about physical activity in this regard, that you have the energy to move, you say?

Yes.

- Yes and can you elaborate on that a little, what do you mean?

It's hard to describe but it is, many people get, they get short of breath and just can't cope, they get tired quickly and they can't move around much.

- And how do you think about your situation, how do you feel about your heart failure and physical activity, is that the case for you?

No, I have, I'm doing very well because I can move and I can walk a lot. It's just that if I go too fast and stuff like that, I have to stop and rest and go uphill and stuff like that.

- Yeah okay, so that's what you have, you get to stop and rest then when you go up hills?

Yes, exactly, but I probably have it very mildly because I can move around a lot.

- You can move around a lot, hm.

Yes.

- You know what, I think I'll change, can you just wait a bit and I'll change rooms.

Yes.

- No, I'm still sitting here and then I wonder, what made you want to participate in this research project, what made you want to participate?

Yes, they called and asked if I wanted to and if I could be of any help to anyone, I'd be happy to come along and tell you how everything is for me, yes.

- So you got a little bit, you got a call there and thought it could help you, did you think so?

If I could help, yes.

- If you could help, hm.

Yes, yes.

- And then I wonder, did you have any expectations before participating in the study?

No, I didn't.

- No expectations?

No.

- No, none at all?

No.

- No, then I'll come to some questions here about the activity coach itself, this stick figure that you would press and move a little, you would type in and then I'm wondering a little bit, what experiences do you have of using this activity coach?

No, I have no experience, I had, before that stick figure, I had to weigh myself.

- But if you're thinking about this stick figure right now because you have some experience, then I'm wondering, can you tell me a little about what experiences you've had?

Yes, what is the answer to that?

- What did you think, did the activity coach affect you?

No, I don't think so. It's just a matter of seeing how much you can handle on different days.

- And what did you think of it then?

Yes, it's always useful to get it in black and white as much as you can.

- Yes, because that's what you saw when you typed in?

Yes, exactly.

- And has the activity coach affected you, has it affected you?

No, I walk my rounds and with my dog so it's like, I haven't walked any less or walked any more, it's just that I can handle my rounds.

- So you're saying, what you're saying is that you 've still done what you usually do and then you've included this in this activity coach?

Yes, that's exactly what I did, yes.

- So I wonder, has using the activity coach affected your physical activity?

No, I don't think so.

- No, you haven't changed your way of moving since you got this on your plate?

No, I haven't, no.

- So I wonder if you had any negative experiences using this activity coach?

No, I don't think so, no, absolutely not.

- No, it wasn't anything you thought about, nothing that was difficult or anything that you thought didn't work?

No, absolutely not.

- And then I wonder if you had any positive experiences using this stick figure, the activity coach?

No, it's that you, yes, you get it in black and white how much you move.

- And did you use it then, did you see it then, how did you do it and see it?

Yes, man ... you keyed in how long , for example, walks you take, so then you get it, that way you get to see it.

- And there was nothing strange or anything like that that you thought?

No, I don't think so.

- Then I'm wondering how you experienced registering physical activity via this stick figure, what was it like?

Yes, it was very, it was easy, I think, yes.

- Can you just elaborate a little on what you mean when you think it was easy?

Well, it was just keeping track of, for example , how, when I went out and how long I was out and then when I came in I keyed in how many minutes I had been out and walking and there was no difficulty at all with that.

- There were no difficulties at all, no there was nothing like, no, no. And then I wonder a little bit, every week your activity was summarized on this screen and you could set a goal for the coming week, how did you experience that?

Yes .. I did kind of in.. I had, the only thing that I, the goal I had was like that I could handle it, not to deteriorate that yes, what I had set as a goal was that it wouldn't deteriorate but it stayed where it was supposed to.

- And then you think about physical activity?

Yes.

- And had you typed that in as a goal?

I don't remember, but I almost must have done that, yes.

- And did you write down any goals then, you may not remember them. Can you tell me more about it, you say yourself that you did not want to deteriorate in your physical activity?

Yes, there was a goal there and then it was interesting to see every week that if I had, if I had walked as much as I had set out to walk, that I could.

- And what is it called, because what you, what I think you're talking about could be this history tab because there was a tab in this screen, on the screen that could be about history, did you use it?

No, I didn't.

- Because you say you looked at the goals every week and it wasn't via this history tab then?

No, but it came up , every week it came up that the goal was so and so and I had achieved so and so.

- Yes, exactly, so you got it right on the screen there, so you looked in this history tab?

No, I didn't.

- Not at all?

No.

- No, no so you have no experience with this history tab at all no?

No, I don't have that.

- Did you use the activity coach in any other ways than the ones we are talking about or that Andreas talked to you about?

No, it was the pedometer that I used then, otherwise it was the same week after week.

- Yes, did you have the pedometer on you then?

I had a pedometer on the first week I got it there, and the last week too. The first and last week.

- There were the tests before and after, yes, but this part is about the stick figure itself. Was there anything else you used, if you used it in any other way, the activity coach?

No.

- No other ways?

No.

- And how much did you use this activity coach?

Yes, every day.

- You wrote in every day?

I wrote in every day, yes.

- Did you do that, Monday to Sunday?

Yes.

- Oh, was it several times a day?

Yes, every time I had been out and about, yes.

- And did you experience it as a lot or a little?

No, I think it was a little.

- What do you mean then?

Yes, I only took my walks about 3 times a day and those were the 3 times I wrote in.

- So you thought it was a little?

Yes, it was no problem.

- It wasn't anything difficult, no.

No.

- And then I wonder, had you intended to use it that much, was that pretty much how you did it, was that how you intended to use it?

Yes, that's how I was instructed to use it.

- Exactly that and how were the instructions?

Well, that was easy to understand , yes.

- So I understand you've been using it every day.

Yes, I have.

- And then again the next question is also about, it's all about this activity coach and I wonder if you saw anything that you should develop... that we should develop with this activity coach to make it better?

No, no, I can't.

- No, nothing that you can think of that would make it even better?

No.

- No, was there something that worked less well?

No, everything works great, yes.

- Was there something you were missing?

No, no.

- No, and then I wonder if there could have been something that would have made you use it more, what would have made you want to use it more?

No, not what I can think of or...

- No, and if you were now offered to continue using this activity coach, how would you feel about it?

Yes, no, I think what I've done is enough.

- And can you tell me a little bit about why that's enough for you right now?

No it is, well I don't know. It's probably awkward.

- And would you like to tell me a little bit about why you find it difficult?

No, it's awkward, but it's a moment that you shouldn't forget because it certainly happens that you've been out and about and then you realize, that's exactly what I haven't done.

- No, exactly that, and was it possible for you to do it afterwards or how did it work?

Yes, I could do that every day, but not, for example, if I had forgotten a day, I couldn't go back and, no, register it.

- So what do you think about the fact that you couldn't do it maybe once a week or what do you think about what you're saying, that you couldn't go in the next day and do it the next day?

No, I don't, if you do it the next day or something then it's easy to, well, you just forget.

- Yes, you mean you forget about it completely, then what do you think?

Yes, exactly.

- Yes, so this that you wouldn't want the activity coach now, can you just elaborate on that a little more I feel, there's a little more I'd like to know about why you wouldn't want it, why you feel the way you do?

No, because I think I feel so healthy and ready for that right now.

- Then I'd just like to hear if you have anything else you've reflected on or highlighted or told us about regarding this activity coach?

No, I think I've done what I've been told and can, so I don't know anything more.

- Then I think I'll end the interview now, we can just stay on the phone and I'll turn off the recording.
