# Supplementary material for: Exploring user experience: A qualitative analysis of the use of a physical activity support app for people with heart failure
Source: PLoS One. 2025 May 22;20(5):e0309577. doi: 10.1371/journal.pone.0309577 (PMC12097600; doi:10.1371/journal.pone.0309577)
Supplement: S1 File — English_verbatim. (ZIP) [file pone.0309577.s001.zip › English_verbatim/BRFL069_eng.docx]

**BRFL069**

- Then it's this activity coach we're going to talk about if I'm going to ask you a little bit about how it's been. You recognize this stick figure when I say that, right?

I can't hear.

- This stick figure you know that was on the screen that was this one, where you were supposed to ..

Yes, exactly, yes.

- .. writing down all your activities, that's what the interview is about here.

Yes, yes.

- And then I first wonder, would you like to tell me what it means to be physically active, what does physical activity mean to you?

Yes, hello?

- Yes, can you hear me?

No, you disappeared.

- Can you hear me now?

Yes, a little better.

- Well then I wonder, what does physical activity mean to you?

Yes, no, I don't really hear..

- Can't you hear what I'm saying? Can you hear me now? Hello?

I have a hearing aid so I have to get used to it.

- Sorry, what did you say?

No.

- Can't you hear me?

Now I hear you, now I hear you.

- Can you hear me now?

Yes.

- Okay, I thought I'd ask a little bit, what does physical activity mean to you?

I'm nobody, I've been very active before but it's bad now because I get out of breath very quickly if I walk too much or move too much.

- Yes, that's right, so you get short of breath when you move too much?

Yes, I can be outside and say if I walk, yes, 2 blocks, half an hour max at a leisurely pace.

- Yes, at a leisurely pace, yes.

It's not working anymore, I feel that, but it's gotten better than it was before, it has, but I find it unpleasant when I kind of get that heart palpitations and have a little difficulty breathing.

- Yes, I understand.

So before I did a lot of sports but now I don't.

- You don't do that now, no. What could it be like, what examples, what could be physical activity for you, do you have any examples of what is physical activity for you?

Now or before?

- No, now I think about it, now?

Yes, it's just a little gymnastic movements for myself, I can do them and lift my legs and arms and all that stuff, that's the only thing.

- Yes, you lift a little, a little gymnastics, you say?

Yes, it is with a little kneeling and getting up and down from the chair.

- Yes, exactly that, exactly that.

That's the kind of thing that osteoarthritis patients usually have.

- Exactly. Then I'm wondering a little bit, you have your heart failure and you also have these symptoms that you describe. They are perhaps symptoms of the heart failure, among other things, from time to time I wonder how you think about physical activity in relation to your heart failure?

Yes, I don't know, no, I don't know, no. So I can feel sometimes it's strange, even when I'm sitting still, it can kind of stick out and feel like something's not right, breathing or I try and sit very comfortably and then maybe it goes away. It's not for long periods of time, it's not, but it reminds me sometimes.

- Yes, it does, yes, and you described before that you get short of breath, you say?

Yes, I will, yes, and of course, when I walk outside, I use a walker because I feel, I got it as support when I had problems with one hip so I got a little bit of this osteoarthritis they call it, and I feel that I walk better with it and I maybe get less out of breath when I walk with it because I lean my weight on it then too.

- Yes, that's right, you can use it a little, right?

Yes, yes.

- What made you participate in this research study?

Sorry?

- What made you participate in this research study?

It was my nurse at the health center. Her name was Birgitta S. She had contact with, yes, it was, her name was Maria, what was her name?

- Yes, it doesn't matter, I know who you mean, yes, that's right.

And so that way and then I started with this but then Birigitta , the nurse, moved so I no longer have that contact.

- No, okay, no. So it was actually the nurse who made you want to be in the study?

Yes, she was the one who thought I should and who wondered if I wanted to take that log and keep track of it myself like this, and it's clear that it's actually been very good because by keeping my body hydrated, it's also kept my weight in check.

- And now it's a little more about this, this specific part of the activity..

Yes, I understand.

- And then I wonder, what expectations did you have before participating in the study?

Yes, I was curious about it and then I thought, yes, but it's probably not that bad and since I had this stick figure, I was maybe a little more careful when it came to moving around and not sitting too much.

- No, so you were a little curious about it and ...

Yes, it was me.

- .. and maybe you had expectations that you would move a little more?

I have an apartment here that you can walk around and I did that very often because I thought that now I should move properly ..

- How good.

The stick figure made me become a little more organized.

- The stick figure made you become a little more proper?

Yes, and I feel like it was beneficial because before, when I walked a little tall, my legs got tired a little faster, but now I don't.

- What did you say, you were earlier?

My legs are tired, but I'm not really tired now, it's much better now.

- Your legs are better, you say?

Yes, it helped me so that I gained a lot more strength in my muscles in some way.

- So my question is, did this activity coach meet your expectations?

Yes, yes, but I think so, I think so.

- Yes, how about up... was this where you were talking about your legs, among other things?

No, I've been walking a bit today and I thought it felt good.

- Yes, exactly, and then I wonder what your experiences are with using the activity coach, would you like to tell us a little about it?

Yes, what activities do I do?

- No, this one, this stick figure now, what experiences do you have using it?

Yes, I don't know if I'll use it in the future.

- No, but I wonder what it was like now that you've had it at home, what it was like to use it?

Yes it was, it was simple every morning.

- Yes.

?? but then I decided I would try and go out and walk for a little while and so on in the morning with keys(?).

- So it was actually that old man who made you decide to go out and walk?

Yes, really, because I thought, now I have to take care of myself when I'm going to be in this.

- And how has the activity coach influenced you?

Yes, it affects me positively.

- Yes how?

Yes, I feel that I, that I feel that now I have to go out and move. Before then, I might not have felt, no, ugh, I'm still sitting there, but that's gone. The stick figure has made me become a little, yes, think a little more about the need to move.

- Yes, how often, how often did you do this during the day?

That, yes, it's very small portions, but the walk around a couple, three blocks, that's probably once a day.

- Once a day, 3 blocks?

Yes, and then I tried to do as much as possible at home, I do everything except cleaning.

- Yes, you do it, you do everything yourself except cleaning, right?

Yes, I take care of everything else myself, so that's when I start the exercise with that(?)

- And then I wonder, you've said that a bit grandly, but how has using the activity coach affected your physical activity?

Yes, I think it has had a positive impact.

- Is it true that when you say you've used the stick figure and you decided every morning how much you would walk?

Yes, I did?? I also checked my weight at the same time and then when I saw that stick figure I thought, well, now I'm going to go out and exercise today.

- Yes, so it affected you, your physical activity?

Yes, it was keeping an eye on me.

- It kept track of you, yes, exciting, and then I wonder if you had any negative experiences using this activity coach?

No, I didn't, no.

- No negatives?

No, no, no.

- And so I wonder, did you have any positive experiences using this activity coach?

Yes, that was why I felt that by doing all this, I actually felt better.

- Yes and how, can you describe this how you felt better?

I felt like, I felt like I, maybe I could do a little more. I thought it was very uncomfortable then when I got palpitations and stuff like that but when I felt like I started moving more it calmed down somehow and then I became bolder too.

- Okay, so it also affected your courage, you could say?

Yes, you could say that.

- Do you think this will hold up or not?

Yes, I think so, I think so.

- Because you don't have this coach anymore, do you ?

No.

- But yes, exactly, are you still physically active?

Yes, I've been out a bit today..

- But are you thinking the same thing...

.. if it's too hot then I stay still, when it's too hot then I don't feel really good but I take it in the afternoon instead . I did that yesterday for example. Then I was out in the afternoon.

- Yes, but that's good, yes, of course. Then I think about it a little and then I wonder how you experienced it was to register physical activity via this stick figure, how did you experience it?

Yes, that was positive, yes.

- Can you tell me a little more about what it was like?

Oh, that's a bit difficult, I don't know.

- No, but you said it was positive that you, this thing about registering physical activity, it was positive, you said?

Yes, yes, I think so.

- And then I wonder, every week your activity was summarized on the screen and you could also set a goal for the coming week, did you do that and how did you experience it?

Yes??

- Yes, but every week you saw a total, you saw the total, how much activity, you remember that ..

Yes, exactly that, exactly that..

- .. and then I wonder...

.. then I tried to impress upon them that I wanted at least as much one week as I had the previous week.

- Yes, so you did, you set a goal to continue in the same way?

Yeah, I probably didn't check the numbers that carefully so I don't know if I did it ??

- No, that's right, you didn't really look back, no. How did you experience it?

It was a bit difficult there and measuring, like, well, I've been walking for 10 minutes now or what have I done, so maybe there was a bit of cheating there sometimes.

- Yes, you had a bit of difficulty keeping track of time, is that what you mean?

Yes.

- But this, this goal then how did you experience it, can you tell me more about why you did it, this and still set a little goal that you would...

Yes I tried, I didn't keep track of the numbers because I know ? Really increasing but I think I stayed pretty much the same.

- Did it affect your goal setting in any way?

No, I don't know if I can say that it did.

- Then I wonder, you know that you could also go in via a tab called history and then you could see how you had registered activities in previous days, did you ever do that, did you go into that tab?

No, I didn't.

- You didn't.

No.

- No, then I wonder if you used this activity coach in any other ways than the ones we've talked about now?

No, I didn't, I don't think so, no.

- No, not in any other way?

No.

- And then I wonder, how much did you use the activity coach?

Oh, I don't dare, I don't actually know, no, it was difficult.

- Was it every day or several ...

Yes, every day..

- You registered every day or what did you do?

Yes, I did, I did.

- Was it several times a day or?

No, yes, I filled up these 10 minutes . I could do that several times a day.

- Yes, exactly that and you did that?

Yes, I did.

- Yes, how did you experience it, was it a lot or a little, did you think, to do it?

No, it was, sometimes I forgot about it and then it could happen that I thought, oh, I haven't marked that, then it could be 2 times instead at the same time.

- Yes, that's exactly what you thought it was, it was easy, you say?

It wasn't that strange, no...

- And then I wonder ..

?? I've been doing the dishes or I'm doing the food and stuff like that, there were 10 minutes that I was moving, then I marked it. Sometimes I forgot it but then I remembered it so I did it afterwards, so to speak.

- So you did it afterwards, yes, hm.

Yes, yes.

- And how, was it in line with how you had intended to use it?

Yes, it was like Andreas said that I should think about those 10 minutes since I was out walking, so of course there were 3 of those pushes when I was out moving around.

- That's exactly what you picked in 10 minutes at a time there, yes.

Yes, yes, so it was a little different.

- Yes, I understand that, hm. So I'm wondering if you've seen anything that we can develop with this activity coach to make it better?

Yes, I don't know, I don't dare, no, I don't know.

- Was there something that worked less well?

No, for me it was, I mean I wasn't bothered by it or that it was awkward or anything like that , no. I thought it was nice.

- Yes, and what was nice?

Yes, like keeping track and thinking a little bit about what you did and getting something to think about and what is useful to do just that and sit down and like it's very easy to do if you have pain somewhere or something and that's what I thought I'll try and stay away from and that's where I got a little push there.

- You got a kick out of this, you say?

Yes, I think so.

- Was there something you were missing?

No, I don't think so, I had no idea what this was about, so there was nothing I could miss there, no.

- No, so nothing that you've thought about that you've missed or could have done better?

No, no, now I'm curious about it, but maybe you can see if it has had any results.

- I can't see now because I'm not involved in that part at all. You might have to ask Andreas about that, I think.

Yes, I understand.

- I understand that you are curious and want to know about that, but ...

Yes, yes.

- I'm curious, what would have made you want to use it more?

What kind of something?

- What would have made you want to use the activity coach even more?

No, no, I don't think so, no.

- No, and now I wonder, if you were offered to continue using the activity coach, how would you feel about it?

Yeah, I don't know, maybe.

- Yes, would you like to elaborate a little on what it is that makes you hesitate a little?

Yes, yes.

- Why do you say, I'm curious?

Yes, what do you mean?

- But I asked like this, what is it called, if you were offered to continue using, if you were allowed to continue using the activity coach, would you like it, how would you feel about it? Can you hear me? Hello, can you hear me.
- Then we'll continue with the interview. Well, but then I wonder this: if you were offered to continue using the activity coach, how would you feel about it?

Yes, I guess that's the same thing I'm going to do then, you mean?

- Yes, so you may not be part of the study, but you would be allowed to keep the activity coach itself? Would you like that?

Yes, so now I don't have the old man here now, Andreas has taken him.

- Yes, but I mean if you were offered it and were allowed to continue and have it, would you want it?

Yes, if it's the same thing, what should I do then?

- Yes, that's what it will be then?

I can do that if it's beneficial to me.

- Yes, because what I'm asking is that if you weren't in the study, would you want the activity coach?

Yes, I can agree with that.

- You would like that, yes?

Yes.

- Can you say a little bit about what, why you say you would like to, is there any reason why you would want to continue?

Yes, it's if it can kind of make me, and I kept saying, take care of myself a little more, if it kind of has this effect on me not relaxing too much.

- Yes, so you would like to continue and use it, you say?

???

- Anything else?

No, it's just that I think a little bit about my health, so to speak, to the extent that I can handle it, that's it, right ?

- As long as you can, yes.

Yes.

- Is there anything else you would like to reflect on or tell us about the study itself or the activity coach?

No, I haven't, no.

- No, nothing else?

No.

- Then I'll turn off the recording here now.
