# Supplementary material for: Exploring user experience: A qualitative analysis of the use of a physical activity support app for people with heart failure
Source: PLoS One. 2025 May 22;20(5):e0309577. doi: 10.1371/journal.pone.0309577 (PMC12097600; doi:10.1371/journal.pone.0309577)
Supplement: S1 File — English_verbatim. (ZIP) [file pone.0309577.s001.zip › English_verbatim/COAN087_eng.docx]

**COAN087**

- It's true that you have a screen at home that you've had for a long time, right?

Yes, that's right.

- Yes, and then I got this part with the activity coach installed, what, it's been about 3 months now, right?

Yes, that's right, it would take 12 weeks, yes, that's right.

- 12 weeks yes, so it's the new part here that we want to focus on in this interview.

Okay.

- This stick figure, you know what I mean when I say that?

Yes, that uncle, yes.

- That guy, he's called the activity coach, but I mean the stick figure, and I'm just wondering, first question here, if you can tell me a little bit about what physical activity means to you?

For me, that means going out and getting some exercise, but my back hurts so much, so I do what I can.

- Yes, you have back pain.

.. there will be some walking and short walks because I can't walk, it's burning down my legs.

- Okay, so it's troublesome with your back, but physical activity means moving around for you, is that what you said?

Yes, I think you should, so you get a little bit of a break.

- So you get a little bit of a break?

Yes.

- Yes and can you give some examples, even more examples of what might be physical activity for you?

Yes, it's because I walk and then you move around a lot. I'm out fishing a lot so there's a lot of walking, you stand and fish on land and yes, things like that.

- Yes, that's how it is for you, that's why you move around a lot when you fish, yes, I understand that.

Yes, exactly, and then there's the osteoarthritis in the knees, you know, that's what comes with age.

- Yes, I hear you have some problems with both your back and knees.

Absolutely, so the back is probably the worst, it's worn out.

- Yes, we'll all probably end up there a little bit if we live long enough.

Yes, you can, as long as you enjoy life and can walk, it feels good. There are walkers if you can't walk yourself.

- There is, yes, absolutely. And then I'm wondering a little bit, you have your heart failure at the bottom, that's why you're in this study and then I'm going to ask, what do you think about physical activity in relation to having heart failure, these different symptoms?

I don't feel any of that.

- You don't feel any of your heart failure?

No, not like that, sometimes it's like something a little big, it hits you extra hard then you get a little nervous and you get scared, otherwise I don't think about it that much.

- And what about physical activity?

No, I don't think about that. You should move so that your heart gets going a little.

- Exactly, but this idea that you're getting a little cranky, is it in connection with something you're thinking about?

No, no, that's when I feel this thing.

- Yes, exactly that and what do you think about being physically active and having this diagnosis?

Yes, I think, I don't know, I don't think about it. In the beginning it was worse. Now it is, now you live with it so that now, well, I don't think about it so much.

- You don't think about it, no, that's exactly it. In the beginning it was a little worse.

No, it's natural, yes, in the beginning it was, then it was hard, but now you don't think about it because you've had it for so many years.

- Yes, I understand, you're used to it now, yes.

Yes.

- How was it, what made you want to participate in this one, these 3 months now that you've had this stick figure, what made you want to participate in this research project?

Yes, I don't know what it was, but I never say no to something like this if it can be better in the future for others who have the same problem, but I want to be involved. Yes, you also find out a little bit about how you feel yourself.

- So it was 2 parts. You think you want to set up for future..

Yes, for future, what should we say then, needs or for other people.

- And then you talk about having something for your personal part, was that something you were thinking about too?

No, it's mostly about keeping an eye on yourself, so I go to the doctor once a year, check my heart with an EKG and stuff.

- Yes, exactly, did you have any expectations for the study, did you have anything in mind before you started?

No, I didn't think about anything like that, I just listened and tried to do what he told me.

- Andreas yes..

.. what I was supposed to do but I have to admit, I sometimes forgot to press it in. There wasn't much to think about but you forgot it anyway.

- Yes, you forgot, but no specific expectations before you started with this activity coach?

No, not directly, no, it wasn't, it was just yes, I don't know, yes and help with the research going forward.

- Yes, so it was the research that you thought you would help with?

Yes, yes.

- And what was it like using this stick figure, what experiences have you had?

Yes, before I had nothing, nothing.

- No, but now you've been using it for 3 months, what do you think about it?

I think it's good. You should do it more often if you have this, then you have a little more insight into yourself, how much you do and what you do, how you move.

- So you say it's good and then you say you've had a good look at yourself, is there something you want to develop there?

Yeah, I don't know, you tense up and make more effort or do more than just sit when you have that because then you go thinking, I'll take this extra lap around the block so I'll get a line like that..

- Yes, I understand.

.. so you got started, you got started in a completely different way and moved.

- And you did, you got started and moved more, do you think?

Yes I did, it was just that, and I don't want to leave here because then my wife will have to pick up my car if it hurts too much so I walk around the block. It takes 5-10 minutes and I can walk slowly and then I can get home too, but if I walk in the forest here like we have, then I might not be able to walk back. Then she can come and pick me up but there are walkers that say where you can start. I'm not ashamed of it.

- No, I don't think you should do that..

No.

- .. no, it is, I have the next question here which is how the activity coach has affected you and then what I think I hear is that you tense up a little, you make a little effort because, because you have received this, why you are involved in this research, is that understood correctly?

Yes, I think it's good because then you do a little more than just sit. You fight a little more even if it hurts, you know what I mean.

- I understand what you mean, yes, just that you got started a little more.

Yes, precisely because you are forgiven.

- You will be forgiven, yes.

I blame everything on corona.

- Yes, you say that, was that when something happened or not?

Yes, that was it, we were barely out of the house here. The kids went shopping and we did nothing, that's how it is. When you get to this age, you just want to be around a little longer..

- Yes, it's clear that you want to, I understand that. This use of this activity coach, this stick figure, has it affected your physical activity?

Yes to the positive that I want to move more.

- Yes, and is it something because you got rid of this one last week, right?

Yes, he was stupid to come and get it.

- Yes, but is it something that you continue to do anyway now afterwards and..

Yes, like today when we went shopping, I often take an extra trip to the store because then I know, then I get some steps on my pedometer too and then you move more.

- So you have a pedometer now too, right?

Yes, I have it on my phone.

- Yes, exactly, but what, would you have liked to have kept this activity coach, do you think, or what do you think about it?

Yes, I wouldn't mind having one like that and you guys coming and checking in every now and then. That's good then...

- What would be good about that then?

Yes, then you see that you move more, that's what I want to show.

- And you don't think you get that, you have this watch, you say, pedometer, but it's not the same thing, don't you think?

No, it's not, but if you make that line, then you feel like you've done something a little extra.

- Just this pressing this, that's 10 minutes you spend there, right?

Yes, you make a dash there sometimes and then you're happy.

- Then you become happy and you don't do that in the same way in the watch, then?

No, then you just see that you have taken so many steps.

- Yes, that's right, no one shouts hurray when you've done it.

No, it doesn't, but you don't move much, but he does move back and forth when you press.

- So this is exactly what I think it sounds like, printing and seeing for yourself.

Yes, it's positive to have it.

- Did you experience anything negative from using this activity coach?

No, nothing.

- No negative aspects?

No, nothing, nothing.

- Nothing no and do you have any positive experiences, any more positive experiences from using the activity coach?

No, it's a bit hard to say now, but it was a bit of a hassle weighing myself every morning, you see, even though I haven't gained any weight. I stick to the same thing all the time, so that's good.

- Exactly that and that's good, exactly that. But you have the activity coach because you've told me a little bit that you still get a little bit, you tense up a little bit more, take in a little bit more...

Yes, I do.

- .. and activates you a little more, but is there anything else you're thinking about, especially about the stick figure you used, anything else you've had positive experiences with that you'd like to share with us?

No, not like this directly.

- Then I wonder a little bit, what was it like to register physical activity via this stick figure?

It was good. I tried to do that, sometimes my wife would tell me, now you have to push, you've been doing something, yes I did it then but then you forgot about it. You sat down, yes now I'm going, then you forgot about it ??

- But you remembered it, did you do it every day?

Yes, I did. We were away for a day and we couldn't do anything, but otherwise, I did every day. Every time I did something, I did it. There were a few times I missed it.

- Yes, exactly, but did you do anything afterwards, did you register afterwards when you had forgotten it or how did it go?

No I didn't, I couldn't take it the next day..

- No, exactly that.

No, I didn't just do it because it's wrong.

- I don't think that's possible either.

No, I don't think so either.

- No, but you still thought it felt, it was perfectly okay to register on this screen, that..

Absolutely.

- ..was there anything you thought of that you would like to share with us?

No, I think it was just positive, you saw when it got to that line, it felt good.

- What do you mean by getting up to this line?

Did it when you pressed(?) on the old man, then a line or something would come up. Then you knew you had done something.

- Yes, it came up, yes, you saw on the screen that something had happened?

Yes, of course, that was good.

- You thought that was good, yes.

Yes.

- What was it that made it feel good, can you just elaborate on that a little?

Yes, I don't know, it felt, then you feel, what I felt was that yes, now you have done something instead of doing nothing at all, not doing anything, you had gotten involved in some way even if it was just a little bit.

- So there was something you saw there, yes.

Yes, it was something, yes, back then you could see that you had done something during the day. Now you can see nothing more than that I had taken a few steps.

- Yes, I understand, yes, I understand, and it was like every week this was summarized, your activities on the screen, did you ever see that?

Yes, it has increased, you mean it has increased a little every day or every week.

- Did you do it yourself or?

Yes.

- So you could set a goal then or how did that work?

Yes, you could do that yourself. It said, something came up, this week you've done so much and the goal was so-so, you increased it or stayed on the same line.

- How did you experience it because it sounds like you increased a little bit week by week or is that true?

Yes, I increased it a little, I did it according to how I felt with my back, but I thought it was good, it was positive.

- And what was good about being able to increase then, can you elaborate on that and tell us a little more about it?

Yes, but then you feel like you were a little more active, you did a little more useful things then or what should I say, yes.

- Yes, tell me more, anything else you want to say there?

No, I just thought, it was a little bit, that you felt more useful then, you beat yourself up, pat yourself on the back, you've done a little extra.

- So this was where you grew a little bit and then you saw these goals in front of you and you achieved the goals or how did that go?

Yes, I did, I did.

- Did you?

Yes, but then we go fishing in August, you see, then I would have one like that because then there would be a lot of steps. We fish from shore so that would be a lot.

- And so I'm wondering, there was a tab right there in the activity coach called history?

Yes, I was barely there, not even once.

- Were you ever inside?

Yes, I looked at it once. I don't remember what it looked like there.

- No, because then you could see your activity on previous days and you could also see weekly.

Yes, at first I went in and looked at that, but then I didn't go in any further.

- What did you think of the little you did?

Well, but that's because... it could be good to go in and see how things were, what the curve looked like, so to speak.

- And what do you think is good about it?

Then you see if you move more, yes or, yes, become more active.

- But you weren't there and saw it like that, or it was a little early, you said?

Yes, that's what I looked at at first, then it became more...

- How come you didn't do it later, was there something specific..

Yes, I'm asking myself that too now that you mention it.

- Because that's what I was a little curious about now, why you didn't do it later, was there something that, could there be something that made it easier for you or have you thought about it?

No, it was nothing like that, it was nothing like that. I don't know what it was, I guess I just didn't think about it anymore.

- No, it wasn't necessary, but I'm just curious.

No, I don't think so, if he had told me there that I would, I probably would have done it.

- Yes, you say that if you had been told yes, but you were still in there and watched a little and scored goal after goal week after week, so you had some control over it, does it sound like?

Yes, I had.

- And patted yourself on the back, you said?

Yes, it was a little pat sometimes.

- So how did it feel then?

Yes it was good, it felt good, no it was good.

- I'm wondering, did you use this activity coach, the stick figure, in any other ways than just recording day by day?

No, I actually didn't, I just went in and looked at it.

- You went in and pressed...

Yes, I pressed the button.

- Yes, exactly that, and then the question comes, how much did you use the activity coach, this stick figure, was it, you said something before, how often did you use it?

Yes, every time I went out and walked or did something, I used it.

- How many times a week was that?

Yes, I can see here, I don't know, it was probably 10 minutes, that's it, I probably had 150 minutes 15 x 16, well then I missed a few, it wasn't that much.

- Were you inside every day?

Yes, it was me.

- Was that you?

Yes, and keyed in when I did something.

- Did you think it was a lot or a little?

No, I thought I did too little.

- Yes, what do you mean then?

That I was too active, I should have done more.

- Yeah, okay.

When you get back pain, when you feel, now I'm just whining, then you kind of give up and have to sit for a while. If you keep walking then it's like, I don't know, it goes down into your legs too so then it gets a bit hard, right?

- Yes, I understand exactly what I do.

But I'm struggling.

- Yes, it sounds like, I hear you do it. You've been great, you've been in every single day on this and registered, it's fantastic.

Yes, I have.

- I think so, was it roughly in line with how you had thought about using this stick figure, did you have any goal with it before then?

No, nothing, but I started up and then I increased, in the beginning I increased every, the first few weeks a little bit so I would, yes, I kept up, I was never below my goal, at least.

- No, because you increased, but did you increase for all 12 weeks?

Towards the end I was probably at the same level but otherwise I increased by one every week.

- And that was pretty much what you had in mind or did you have any plan before there or you..

No, I had no plan at all, I just did it for my own sake, I would see.

- And then I wonder, can you see anything that we could develop with this activity coach to make it better?

But I think it's good if you use it like, when you've done those 10 minutes and make a dash there. I think it's good because, then it's up to each person to remember and press dashes too.

- Yes, can we do something better there?

No, I don't think so. I think it's well developed as it is, as I saw it.

- Was there something that worked less well?

No, it worked well, you weighed yourself in the morning and then you looked there and then when you had done something you pressed the button and it was registered, you then removed it, no, it worked well.

- Do you think there was something missing?

No, I don't think so. It was simple and good.

- How great, was there anything that would have made you want to use it more, could you, could there be something that would have helped you and use it even more?

Well, I don't think so, but I thought it was good. It was simple and good.

- Simple and good and now it comes, you said that answer a little earlier, but if you were now offered and continued to have this activity coach, how would you feel, how would you look at it?

Yes, I would only see that as a positive.

- And then I want to know why?

Yes, I would like to know so that you can, put a little more energy into it and move more. That's what's wrong when you don't have anything, you take it a little too far then you sit but if you have something like that then you can, you become more motivated if I may say so.

- Motivated to?

Yes, move more.

- Move more, anything else that you think you would like to continue and have?

No, I don't know, well that's the purpose and being able to move forward if you come up with something good for this heart fibrillation, so I'm happy to support that in such cases.

- Then I wonder if you have anything else you want to reflect on or if there is anything else you have thought about regarding this stick figure, the activity coach, before we finish?

No, nothing, I just want it to start moving upwards more so you can see that I've moved more.

- You can keep going and going and going and going.

Yes exactly, yes but if you have one like that if you start at 4 times a week and then yes but this went well, then you take it next week then you take it 6 times maybe so I was at 15-20 maybe and then finally after a week or so then maybe you're up to 10 instead of 4 from the beginning. Then you see, then you feel good inside yourself if you say so.

- You feel good inside, yes.

Yes, then you know you've done something.

- Yes, exactly, anything else you want to say before we wrap up?

No, I think you're doing a good job actually working on this. This needs to happen, yes, move forward, and you've done that quite a bit, actually.

- Yes, it sounds like that, now I'm on the third interview here and it sounds a bit like what you're talking about, it's a bit in line with the others. But then I'll turn off the tape recorder now.
