# Supplementary material for: Exploring user experience: A qualitative analysis of the use of a physical activity support app for people with heart failure
Source: PLoS One. 2025 May 22;20(5):e0309577. doi: 10.1371/journal.pone.0309577 (PMC12097600; doi:10.1371/journal.pone.0309577)
Supplement: S1 File — English_verbatim. (ZIP) [file pone.0309577.s001.zip › English_verbatim/EIAR027_eng.docx]

**EIAR027**

- Then I would first like to ask you to tell me, what does physical activity mean to you?

Yes, physical activity is that I walk with a high wheelchair like this called a dog board or something, a wheelchair I think they call it, which I have to walk because I have problems with my back and with mucus in my lungs, so I have to walk so that I can get the mucus out a little better and that my back can stretch out so that I can walk every single day, I walk. That's my physical activity. Then I have a little bit that I pull the arms that hang on the door to my bathroom, I hang, like I have to pull my shoulders.

- Yeah...

Because of them, I had surgery so that I have prostheses like this in my shoulders.

- Exactly, exactly, could you give any other examples of what could be physical activity that you can think of?

Well, it's when I go out, come out on our big veranda here that I can go with that and do that gymnastics, what's it called, the activity with the chair, the high wheelchair, so that's it, I get out in the fresh air so that I get fresh air into my lungs and that. That's what I can say. I don't have much else, I mostly sit inside.

- Yes, yes, tell us a little about how you think about physical activity in relation to having heart failure and symptoms related to heart failure?

Yes, but it's hard because as soon as I get to use my arms, I get short of breath and don't feel so good, so here, I have to keep getting dressed and keep my arms up, so it's hard because after the heart surgery I got that heart failure and then I also have kidney failure, so there's a balancing act with the fluid that I have to take in.

- Exactly.

So I probably have to tell the doctors that I need to have 1½ liters more fluid for the kidneys, but then you shouldn't have that much fluid for heart failure, but it turns out that I have to think about the kidneys too.

- Yes, and these symptoms that you have from the heart failure, can you relate, it affects how you relate to physical activity. You said yourself that when you use your arms you get short of breath?

Yes, exactly that, hm, no, but it's true that I've been, I'll say, as soon as it has to do with the upper part of the body, it becomes a problem, I can get regurgitation of mucus and stuff like that, I get it all the time. That's what's hard for the heart too, that I can't tell when it comes, but it comes all of a sudden , and then I use that scale with this plate like we do, so that my daughter knows how much medicine she should give me for the heart failure here? ?. It can change from day to day, so the weight.

- What made you decide to participate in this research project?

Yes, I've wanted to know(?) a little more about this disease, primarily, to get some information about these restless legs that I have and they've actually gotten worse lately so that it's been hard sometimes in the evenings and nights and you wake up because they're moving.

- Exactly, ugh.

So unfortunately, when you get this old, you get more diseases as you get older.

- What expectations did you have before participating in the study?

Yes, it was because of this with the medications that it was so that I had the right kind of medication and so on. That was primarily what I thought about this with Sifrol and that was it? There is something else too but I have had it for so long, I have had it, for almost 20 years this medication and then the doctor has removed some medications, this doctor that I had that I had before but I have told her so that now I have another one. I don't know, they don't know much about this with restless legs I think the doctors, the young doctors I think it seems like.

- Exactly, did you feel that these expectations were met?

Yes, yes, they were fulfilled more than I thought, in fact, since you contacted me, it was actually more than I thought it would be. I just thought you would have some statistics or something that you would look at, but it was fun that I got to be included in this survey.

- Hm, then let's go into a little more detail about what this study was about. You were allowed to use this tool that I call the activity coach, where you could indicate how much physical activity you did and so on. Can you tell us a little about your experiences using it?

Yes, it wasn't a problem at all. It was just that I had heart surgery, so I've been through a lot, so I thought it wasn't a problem at all. I didn't think it was there, I just made sure I put it on every morning when they dressed me and that. It was just what it was, I thought about it, otherwise it was natural, I think.

- How would you say the activity coach, as I call it, has influenced you?

Yes, it's like I said, it hasn't really affected me in any way, it hasn't. It's just that it's going to be interesting to hear what it showed, that's the only thing because I'm a little curious about the layout so I think it would be fun to see if? Was it something like the others or if it was something special.

- How has using this activity coach affected your physical activity?

No, it hasn't actually affected me at all. I've only worn it around my stomach or around me, so that it hasn't bothered me or in any way, without it being almost like a workout garment, I could say.

- But did you have any negative experiences using the activity coach?

No, nothing negative at all, I've had so many surgeries and I've had about 15 surgeries so that's why I'm used to different activities like this and I've had to do gymnastics after heart surgery and everything like that and the same when I had the herniated disc surgery and I've done gymnastics or had a lot of activity they call it now so there's been a lot of that stuff I've had. The only thing is now that I walk with this big walker and that I'm working on my shoulders with this one that I'm going to move with.

- Exactly, did you have any positive experiences from this attempt?

Yes, I thought it was nice to talk to you. I think it was positive and that there was someone who really cared about this sick thing, this restless thing that I have too, that someone knows about it because there are many, they notice it when they put my compression stockings on me that my legs jump and go like that.

- How did you experience registering this physical activity with these pluses and minuses that you did when filling this can? Do you have any memories of how you experienced it?

No, it was probably just that I got, I thought I was filling out a piece of paper like I usually do, these kinds of surveys and stuff like that so it wasn't anything special or difficult in any way, it was just that it was, I wrote down what I felt like so and unfortunately I've forgotten what was on that paper because I don't have a copy of it. Since I've been a secretary now, I'm used to having a lot of paper. Back then, you had copies and stuff like that , so you miss that, but it's the computer that matters. That was already the case in my time, but unfortunately, you're still living in the old way.

- During the 12 weeks that you had this equipment at home, your activity was summarized on this screen each week and it showed how much you had moved last week and you could also set a goal for the coming week. How did you experience that?

Yes, I don't remember that, unfortunately this recent memory is actually playing tricks on me so I don't remember it, I remember it because it's like this in the near future, the months it kind of gets forgotten when you're this old, unfortunately that's how it is.

- There's another question about that and then you might not remember it but I'll ask it anyway so that I have asked it and on this screen there is a history tab where you can see, for example, how your weight has varied. On that you could also see how this, how much physical activity you registered in the previous days, do you have any experiences with it that you can tell us about?

I haven't actually thought about that, well I go back and look at the history there I do, we go back and look at, my daughter and I but I haven't reacted to it being so different, I haven't actually thought about that but of course it has to be because it's different days. I've gotten worse with this heart thing and the fact that I have more feelings about it now so that, you're not supposed to live forever either.

- No, that's certainly true, but ...

But I don't take it too seriously, but I'm really happy that I have this so that my daughter can check her medications with this screen, that's how I weigh myself every day. I can miss a day and the girls who forget it also have the home care workers who help me because it's hard and standing on that because the scale sways a little when you get up, but otherwise I don't think it's a problem. It's about the same weight I have, it's just a few kilos different. It hasn't been anything different.

- Did you use the activity coach that we are talking about in any other ways than what we have talked about so far?

No, it's just that I look back at different months, weeks and so on what's on that screen and I never go back further than a few weeks so that but it's a very good tool because you have this thing with heart failure and different things on your legs, so some days they're very swollen and stuff like that you have to fill in on that screen and so that, no, we use that screen a lot actually.

- That was the next question then, how much did you use the activity coach, so how often?

Yes, it's every morning, we can miss a day a week, but it's at most one day a week and it's not often that we miss it, it's probably every day at most.

- Did you experience, or do you experience it as a lot or a little?

No, I take it as a habit that I'm going to do that, it's my habit that I'm going to do that, so I don't think it's a lot, but I actually think it's very good.

- And would you say that it was pretty much how you had intended to use it as well or did you have other expectations?

No, I probably understood that it would be like that, that I would use it like that.

- And then it might be a little more, what should I say, speculative or if we look ahead a little bit. From your perspective, do you see anything that should be developed with this activity coach to make it better and there is no right or wrong answer here but I'm just looking for your personal opinion?

Yes, I think about it, that question came so quickly, I have to think about it. I have, of course you know that when you stand on the scale and like this you have other thoughts, then afterwards they have disappeared. Unfortunately, age is a tick in the bank for me but yes, no but what is it called, I think that it, we just have, both my daughter and I, we only have positive things to say about it so that she can change the medication and remove a tablet or also add a tablet thanks to the fact that I have weighed myself this way and that there is a date on this scale, akti .. yes what is it called?

- Yes we can, the screen maybe hm.

The screen, that's why it's great that it's stored here.

- Was there anything that worked less well in your opinion?

Yes, it's this, that you're worried that it won't show up on the screen, that I've had a little problem with it, that the battery or something like that was down and the last time here when the girl came and was going to fix it because I had called that it didn't show anything on the screen, it only showed the scale, then she just took it and I don't know what she did, she just held it in her hand and did some stuff because she didn't do anything to it and then it started up so it's lucky that you have those girls who can come home and help you and get it sorted.

- Yes, but how good.

Because it was down for almost 14 days, I think, because it wasn't working and it was the weekend and it was the Easter weekend.

- Was there something you were missing?

I would have liked to fill out those questions in the peace and quiet of my home, not on the phone.

- No, I understand.

?and remember like this.

- Is there anything, what would have made you want to use this physical activity recording even more, do you think?

Yes, but it's because I feel that the mucus that I have in my lungs ... down in my lungs, that it comes up better and so that I don't have to cough so much from it so that, and that I haven't had to bring up, well bring up food and mucus like I had a while ago but we don't know if it has to do with stress and also doing that, that you feel stressed, I don't know but no I think it works very well otherwise when it works and that's what it does most of the time, it works.

- If you were offered the opportunity to continue using the activity coach, how would you feel about it?

Yes, it doesn't matter, it's going well. I have nothing against it because when you have heart surgery, you have been through so much and as I said, I have been operated on many times with herniated discs and everything so I have been through and shoulders here, the shoulders are this prosthesis that I have, there all the nerve fibers go through up to the brain through the shoulders so that, he explained it to that doctor that I had at Danderyd, he was a very good doctor I had there. I have had very good doctors all the time.

- Yes, that's fine.

Except for her who went on all the medications, but that's another story. I'm allowed to say that I am, I have nothing against it.

- Exactly.

If you send someone like that, then there's no problem that I can wear it.

- Exactly, then the last question I have here in this slightly more formal interview is, if you have anything else that you want to reflect on or highlight or talk about generally connected to physical activity, this activity coach and registering activity and so on?

Yes, that's what I'm talking about, how to get my legs better, so they don't jump and fall on their own. It's a ?? and there might be a point on this screen about some other problems, not just the heart, maybe a point like that , I don't know if it's possible to bring it in or, that's the only thing I can think of. Now I've got a little problem with my head here. I don't know if you saw that I had a wound on my head.

- Yes, I remember you telling me that.

Now I'm waiting for them to hear back so they can do something about it because it's so basic, what's it called, basic, it's a kind of cancer.

- Yeah, ugh then.

Yes, but it's this kind of mild cancer, they say , ? That kind, a milder kind, but it has to be treated.

- Yes, yes, but then I hope you get help with that in every way.

Yes, of course, I hope so too.

- I'm just going to take and finish the recording so that this formal part...
