# Supplementary material for: Exploring user experience: A qualitative analysis of the use of a physical activity support app for people with heart failure
Source: PLoS One. 2025 May 22;20(5):e0309577. doi: 10.1371/journal.pone.0309577 (PMC12097600; doi:10.1371/journal.pone.0309577)
Supplement: S1 File — English_verbatim. (ZIP) [file pone.0309577.s001.zip › English_verbatim/INSE098_eng.docx]

**INSE098**

- We have clarified that it is this newer part in this, on this screen, the activity coach that the interview will be about and what is it called, I'm just wondering in general terms first, what does physical activity mean to you?

Yes, it includes everything from walking to a little more intense stuff, so to speak. Not that I do that much, but for me, a regular walk is physical activity .

- It's for you.

.. and then yes to some extent it's vacuuming and window washing even though you only use your hands maybe when washing windows and your legs but vacuuming is a bit more. It's not the same as vigorous exercise if you say, interval training and things like that, that's more for me.

- And for you, I hear that you're out walking and even cleaning and window cleaning can be physical activity for you?

Yes, I think so, and gardening above all. I think it's very tiring these days, anyway. It's much more tiring than walking, really, if you just walk mostly on flat ground. Of course, you walk in the woods and fields, which I do quite a bit, or mostly almost, then maybe you can compare it to the uphills, anyway, but on regular flat ground, gardening is much more tiring, I think.

- And then we get into a bit of a dilemma because you have been diagnosed with heart failure, right?

Yes.

- And then we think in relation to your heart failure, how do you think about physical activity then?

I know that it is very important to move every day if you say more or less, so I try to do that, but sometimes I get careless. I still move, but I mean I might not do anything more strenuous. I might just walk at home in the worst case if you say I can't go out, but most of the time we go out, because I feel I have to, because I have always moved, so it's not just because I have heart failure, but it's still there. You're used to being outside a lot and walking a lot.

- And do you have any symptoms that affect your physical activity?

Yes, it's that I don't have as much energy and that I get short of breath, it bothers me a bit.

- It bothers you a little, yes.

Yes, I think that's hard. It's hard to accept, but that's how it is, and then it's age, too, I understand, but I blame the heart failure. You have to have something to blame.

- Yes, you have to, yes, so you can feel that you are still a bit limited then?

Yes, I think so, I don't like to run unless I have to and I think I'm really clumsy and jump and stuff like that so I shouldn't do that because it 's not good for me, say those who know, but it bothers me a little that I don't, I don't jump like I used to.

- Now I'm so curious and jumpy, what are you thinking?

Yes, but then I think about what I jumped when you do some gymnastics and such and jump rope, for example, it's very difficult, I think.

- Yes, because then you will be limited?

Yes, I don't think I can handle it, but it doesn't have to be because of the heart failure, it might just be because you get clumsy with age, I don't know.

- No, yeah, I don't know either.

It's both and maybe.

- Yes, and I'm just wondering, what made you participate in this research project anyway?

Now I didn't really hear.

- What made you want to participate in this, in the research?

No, but I think you should volunteer if it can help someone else, it's more that I feel more or less forced to do that.

- Well, that was a bit of your motivation to volunteer, but did you feel forced?

No, it was me who thought that you should just accept that you are just that way, I think.

- You think, yes, exactly that.

Nothing else, no one forced me.

- No, that's good and I wonder what expectations you had before participating in the study?

Actually, I didn't have any huge expectations, but I thought, I'll do this and we'll see. I know very well how much I need to move and feel good, so I thought about it, I didn't think it would make that much of a difference.

- No, and the expectations you had, did you think they were fulfilled?

I think it was about the same. I think it could be good for some people, especially older people, who move a lot less than I do. I can't say I moved more because I had this, I don't think I did anyway.

- No, so you didn't really have very high expectations and that's pretty much how it turned out, you thought, is that how I should interpret it?

Yes, but I think it can help others if you tell them. Then it's worth something anyway.

- Absolutely, yes absolutely.

I think it depends a bit on how much you move anyway, how you motivate yourself.

- So I'm wondering, would you like to tell me a little about your experiences of what it was like to use this stick figure as we call it?

Yes, I told Andreas that I thought it was difficult, partly to know what an activity is, as I said. I think it was a bit vague, I talked to him about that when he was here and then I think 10 minutes, what about 10 minutes. He said that you could add it up if you did 5 minutes maybe something and then 5 minutes, no I don't know, I thought that was a bit tricky if you say ????

- Did you say pussy?

And then you forget it if you say. One day I wasn't home and then there's nothing and you have to put it on another day or and then mine was very strange because there was something wrong with it when you, you had to fill in how much you wanted if you wanted more next week if you said increase and it started out like so low and then it never changed so it only took up the increase from the previous time not from my increase what numbers I had so I don't know if there was anything strange about it and I told Andreas that but it doesn't really matter to me if you say that.

- But it still sounds like you might have wanted to use that part more?

No, not really, so for my part I can't think that, so it doesn't feel like it would help me at the moment but it's possible that it would when you move a lot less if you say you would have a better overview then if you were to be bedridden and barely move at all. I think it's more for them, that's how it feels to me but I could be wrong there.

- No, but that's what I want to know, how do you think about this now, so that this question about the activity coach has affected you, maybe, has it affected you in any way?

No, I don't actually think so, to be honest.

- No, and you may have said this, but has this stick figure affected your physical activity?

No, I don't think so either, I think it was mostly difficult to know what you had done and how much. It was easy when I was working out because then I knew how long I had been doing it, but otherwise when you're walking, when you take your time, when you remember and take your time, otherwise you had to take your chances and then these little things like vacuuming and window washing and stuff like that, I think you didn't think about how long it took so you had to estimate it a little bit and so you probably took less.

- Yes, okay, yes, so for you it was a bit difficult to define activity?

Yes, I think so, what is activity if you say. That's what you asked at the beginning, people can have very different opinions about it so I think we should continue and have that, I think we should be clearer about what activities are. Then it can be different for different people, I understand, so it can be difficult too.

- This is really interesting because that question comes a little later too and you can, but this is exactly what we want to know, do you know how to and be able to develop and then I also wonder and you maybe said a little bit, did you have any negative experiences from using this activity coach?

No, I don't really think so. It's just one more thing to remember.

- What was it that made it, there was something there that you experienced, you said you would remember it, what was it that you felt then?

Actually, it's that you think you should, you should maybe do it right after you've done the activity, then it's hard to remember what I've done so I, no I know, no, I thought it was a bit messy and sometimes I came up with it after 12 at night and then you couldn't fill it in.

- Yes, it was too late then . Would you have wished you could have filled it out the next day?

Yes, I think that could have been smart actually. That's one aspect of it all.

- It didn't work out now, I understand.

No, it didn't work, no. Then you have to put in that case instead . Then that would be a bit wrong too. In and of itself, it's the week that it's calculated per week so you should, you can actually do that.

- Yes, you can actually do that. Did you have any positive experiences using this coach?

It's possible that you think a little more about feeling like I should do something now, but I think I would have done it anyway if you say so, because I can't say that I feel like I did more because I had it.

- So possibly they felt they should still do it a little grandly.

Yes, maybe you don't want to leave it empty, that's what it is.

- So you felt you didn't want to leave it empty ?

Yes, you feel that there is a certain compulsion, so in that way it can be good.

- Yes, that and the way it could be good, what do you think, can you just elaborate a little on this that you might be afraid, that it could still be good?

Well, but then it's a little incentive maybe that you have that, especially if you have a tendency and a lazy mind if you say it for those who have it, I think so.

- Exactly, because then I'm wondering a little bit, now it's the practical part, how did you experience registering these physical activities via the stick figure, what was it like pressing this stick figure?

Yes, there was no problem with that in and of itself. It was more that and appreciate especially if it was shorter things that you had to add together so I don't think I did much of that really. It became these longer sessions if you say that you knew that you had done this particular small walk, so it doesn't count in any way.

- No, exactly that.

But like if you have a pedometer which I don't have anymore but I mean it doesn't count, for me it doesn't count even if you move, you move all day long really if you're up. Sure you sit sometimes but you're up quite a lot and walk in between ?? if you say so myself then.

- Because it sounds a bit like it was this one and knowing as you said before for you physical activity is something different and for someone else it's something else is where you kind of grand...

Yes..

- Because it doesn't count, you say the steps because you do it anyway, yes.

No, yes, that includes living in some way, if you're not bedridden, you're up quite a lot and moving around all the time, but yes, I can't count those steps if you say so. It gets tricky, you can do that with a pedometer, but you can't do that with this, if you know what I mean.

- If a pedometer had been connected then, what would it have been like?

I think that would have been better. I think that gives you the idea that ordinary steps are considered physical activity, and in a way they are. For me, I think that would have been better.

- That would have been better for you, right?

Yes, I think that would have felt more natural, simpler in some way, I think.

- Then it was a little big on the screen then, you could, you said that before, your activity was summed up each week and you could set goals for the coming week, how was it, it didn't really work for you or did you?

No, yes I did anyway I thought you're allowed to bet a little but no so there was something wrong from the beginning. It just kind of went up from what was the first time so to speak, then it never happened, it went up maybe if I, now I don't remember if it was 10 just that little bit if you say so you didn't get the amount you had the previous week if you say, you understand?

- I understand, it didn't really feel it.

No, so there must have been something strange about that one.

- Yes, exactly.

But I told Andreas that too so that ...

- Okay, you didn't get a new one then ?

No, because I didn't tell you until afterwards. I thought maybe that wasn't that important to me, to me it didn't feel that important because I knew I had...

- But could it have meant anything, do you think, since you started using it?

No, I don't really think so.

- Did you have a goal, did you have a goal, what did you have, what were your goals at the beginning back then, do you remember?

No, it was just that you wrote higher because it was already a sum, like the first calculation, so there was a sum and it was maybe only 70, it was really little for a whole week and then you had to write a higher one and then it was significantly higher then the next week when it was summed up and then I wrote higher but that's how it was from the old one, you understand?

- Yes, I understand, it's a bit frustrating.

Yes, but it didn't really bother me.

- Did you do it, did you give up, or how long did you keep writing goals?

No, that's how I wrote it anyway.

- Yes, you did it, yes.

I thought maybe it would change at some point, but it didn't. But I saw what I had to say.

- Yes you did, yes you did, yes you saw, yes you saw. How did it feel to see that?

It felt completely okay, but it's the same thing there that you say, yes, what was the activity, how does it count and what counts, it was very vague if you say so for me it was difficult to know what should count.

- Yes, I understand, and yet you had a discussion about it, I understand?

Yes, yes absolutely, no I found it difficult to calculate exactly what I should do.

- Yes, I hear that and then I wonder a little bit, now I don't know if it was relevant but there was a tab called history, was it something that you were in and clicked on that tab?

That was probably bad, I think I probably did it at some point.

- Didn't you do that once?

Well, I don't remember if I ever did that.

- Can you remember what it was like then, do you remember what you saw there?

No seriously, I don't remember, no I think I did but I don't remember, no.

- Did you use this activity coach in any way other than ...

Not just that one, but the usual one and weighing yourself and so on ..

Talking at the same time.

- Yes, exactly that part, exactly that and how much did you use this activity coach?

How much?

- Yes, how much, it was like ..

Yes, you mean several times a day ..

- Yes, I wonder how ...

.. yes, but it was when I remembered it, I did it several times. Sometimes I printed in the evening so I could think about what I had done today.

- Yes, that's exactly what you summed up last night.

Yes.

- And was it, did you think it was a lot or was it a little?

What do you mean, a lot or a little?

- I'm more thinking about whether it was like, if you thought it was a lot of work or was it just...

No, it wasn't really, it wasn't a lot of work.

- Whether it was burdensome or whether it was...

No, I don't think it was burdensome, it was more that you wanted to remember it, that's probably why you sometimes didn't remember it, but I can't say it was difficult.

- Could you improve anything about this, remember, can you think of anything that could be improved?

Yeah, what could it be?

- I'm thinking more about those AIs that exist now?

I'm very bad at that.

- Yes, I understand, no, I'm just curious.

Do you mean remind or something more?

- No, I don't know, I'm just asking if you can think of something?

No, if you're going to register it yourself, you have to, or you should be reminded to do it. It might be good to be reminded in the evening so that you at least do it then and don't forget it again.

- No, and how do you think it would be remembered?

No, I don't know, I'm not familiar with that kind of thing.

- No, I think you can think completely freely here. But then I wonder if it was roughly in line with how much you had thought and used this coach that you printed every day, you said, was that roughly what you had thought?

You mean how I had thought before...

- How, use it?

Yes, that was it, I guess you could say that.

- Then I'm wondering if you see anything that we can develop with this activity coach to make it better?

Yes, that's exactly what it is and making it clearer what you should think, so to speak, what you should write down, so to speak, write down.

- Are you thinking about your activity now?

Yes, what is what, so to speak, and then maybe something so that you remember it, but you can maybe, I don't know what you can put in that , but you can maybe do something the next day so that if you forget it, you can go back or something like that . I don't know if that's possible.

- Some kind of reminder, you think?

Yes.

- Was there something that worked less well then, now you may have said a little grand, it didn't really work ..

Yes, that was the setting there, but it didn't bother me, I just registered that it was like that.

- It was more that it was crazy because it was a research study, I think it would have worked.

Yes, but it didn't really bother me, it didn't bother me to do things if you put it that way.

- Was there something you were missing?

No, so I'll be honest so I can say this that for me I can't say that it made any difference . It felt like I didn't get much use out of it but I'm sure others might. That's how it is, it's about the same as I feel about the scale if you say at all that I might as well weigh myself on my own scale and write it down sometimes as long as I feel like I do now and have such a stable weight if you say so, you understand me.

- I understand you very well.

That's how I feel and it was probably the same with this one.

- Yes, I understand, and was there anything that would have made you want to use it more?

No, I can't think of anything there really. It's that thing you were talking about connecting it to a pedometer, but you can use that, fix a pedometer yourself if you want.

- And if you were offered to continue using this activity coach, how would you feel about it?

No, then I think I would say no at this point.

- And by the way, do you want to tell me why?

No, as I said before, I feel that right now I don't need it, that it wouldn't help me if someone told me to feel better.

- And do you have anything else you want to say, reflect on, or tell us about?

No, otherwise it was nice if you say, Andreas called several times so it was nice, he was here and so on. We can only hope that it helps others.

- You know what, yes, I hope so, and then I'm just going to turn off the tape recorder.
