# Supplementary material for: Exploring user experience: A qualitative analysis of the use of a physical activity support app for people with heart failure
Source: PLoS One. 2025 May 22;20(5):e0309577. doi: 10.1371/journal.pone.0309577 (PMC12097600; doi:10.1371/journal.pone.0309577)
Supplement: S1 File — English_verbatim. (ZIP) [file pone.0309577.s001.zip › English_verbatim/KRPH047_eng.docx]

**KRPH047**

- You have this screen at home, right? Because you've failed, right?

But now he brought it with him the other day when he was here.

- The whole screen?

Yes, everything, I said I don't want it, it's been a hassle.

- Yeah.

"I have my wife with me."

- But what was the problem then?

Yes, sometimes it wouldn't work, changed the battery and then it worked??, fiddled with that, no, it wasn't fun.

- But this is for your heart failure and weight, and you haven't had it before, have you?

Well, I've had that partly, but now? They've been out tinkering and helping, but it's been like that, yes.

- Yeah, it hasn't worked?

No.

- No, okay.

??

- Sorry?

I told Andreas to take it with him.

- Yes, he would take it with him, yes.

Yes.

- The question is whether you should remove the speaker and speak into the microphone, let's see if it gets better.

Well?? then we'll see what I can do...

- Turn off your speaker.

Yes, I'm doing it here, but you have to do it that way.

- Let's see if it gets better.

Can't get there, do you have to???

- Now it's better if you speak like that, if you keep your mouth close to it.

Yes, I drive like this.

- Is that okay, okay , but then you had to use this, a part of this screen called activity coach...

Yes, exactly.

- .. and this weight mania .. , when you weighed yourself and stuff like that, that's not really what this interview is about right now.

No.

- Without this, yes this stick figure if you know what I mean?

Yes, yes.

- And I'm just wondering in general now, what does physical activity mean to you?

Yes, it's good to move around.

- Yes, do you want to tell me more?

Well, actually you should go out and exercise a lot, but I'm bad at that, very bad at it and always have been, but on the other hand I do other things and work and... So that's what I do.

- What did you say you were doing, you said?

I work on other things besides exercising. For example, I chop wood and mow the lawn, everything is possible here at home.

- So when you say, when I say physical activity, what do you mean, do you divide it up?

No, I don't, but I just go with my wood when it's relevant and then there's something else there, like, it's going on all the time, basically, and I'm really bad at exercising.

- Yes, that's exactly it and to me it sounds like, when you say exercise what do you mean?

Yes, going out and doing physical activities that pretty much everyone else does, I'm really bad at that.

- Yes, but for you then, for you physical activity can be something else then, can you elaborate on that a little more, you said something just now?

Yes , for example, working with firewood, that's a job called gods, I think? I'm doing some of that, yes, I start early in January and then I'll do something with it until April and then I'll take a little vacation and go for a ?? yes, I'm doing a little bit of everything. And then we have a villa at home here so there's some work there too.

- So what do you do then?

Yes it could be ? And it could be something else with the house or yes digging, yes there is always something . ???

- What did you say?

Rake and make it nice, I said.

- Do you have a large garden?

Yes, but it's probably 1100 square meters so there's always something and I want to do something.

- Exactly that and now you have, I know you have heart failure..

Yes.

- .. and then I'm wondering how you think about physical activity in relation to your heart failure?

Yes, I think I do what I can do, so I'm limited compared to before I had the failure, if I may say so. Then I could keep going as long as I wanted, but now it says no, rest between rounds.

- And what is it, can you describe a little more what is happening?

Yes, it's the energy that runs out, then there's no energy left.

- No.

No. That's pretty much it.

- Yes, and what do you think about physical activity in connection with heart failure?

Yes, that's right, I do what I can and then I go inside and rest.

- Are you resting?

Yes, I'm taking a nap.

- You take a nap.

One like that You know, goofballs .

- Great, do you think that helps, can you continue then?

Oh yes, if only I could rest for an hour then I'd be up and running again.

- Then you're up again, yes.

Yes , ???

- How did you come to want to participate in this research project?

Yes, he called me Andreas and introduced himself and what he stood for, etc. and yes, I thought, okay, I'll do that.

- Yes?

Yes, that's how it is.

- What did you say?

Yes, that's how it is.

- And what expectations did you have before participating in the study?

Yes, that's something I hadn't thought about, but I thought I'd volunteer and then be able to be of some help there.

- What did you say?

Yes, if I can be of any help, I will be.

- Yes, so this thing about expectations, can you think of something now or what ..

That's the question, I probably had no expectations at all, but it was a study for me and yes, it could be positive for everyone.

- Sorry?

That could be positive for everyone.

- Yes and what do you mean by positive when you say that?

Yes, that's good, that it's good that there is research, etc.

- And what do you think is good for?

Yes, the research purpose.

- I'm wondering a little bit about your experiences using this stick figure, would you like to tell us a little about your experiences?

Yes, the stick figure, I just used it and wrote down how I felt and activities and hours and all this , that's what I tried to keep.

- Yes, would you like to tell me how you did it?

Well, I did as the instructions said, filled it in at the end of the day.

- Filled in at the end of the day.

Yes, I am ?? morning

- What did you say?

In the morning.

- Also in the morning?

Yes, I weighed myself.

- You weighed yourself, yes, that's right. And has this activity coach influenced you?

No, I can't say it has, I can't really say that.

- It hasn't affected you at all?

No, no, no, I don't know if I agree with that.

- No, I'm just asking if it has affected you?

No, no, say no there.

- And I wonder, has using this stick figure affected your physical activity?

No, absolutely not, no, I don't think so, no.

- And can you tell me a little more about that, how you think about it?

Well, I took that as a compulsion and filled in all that and then the stick figure was there so that was the only thing, there was nothing negative about it and it went well.

- Yes you say something, did I hear you say coercion?

No, not forced, but? Anyway, no, and then this thing about times and what was, I was out and about, I work almost all day sometimes so I forgot to fill it in but then my wife did it for me.

- So you got help doing that?

Oh my gosh. With my fingers, there would be a lot of stick figures.

- Yes, exactly, did you experience any negative effects or experiences from using this stick figure?

No, no, I can't say that.

- No negatives?

No.

- No and have you had any positive experiences using this coach, the activity coach?

No, no, not really, no, no, it's been just as good without it.

- Yes, can you elaborate on that, what do you mean?

Yes it's a little, no I don't remember but it will come later ???

- How did you experience it?

Yes, it will be a bit difficult then.

- How do you think it was difficult, do you want to tell me more?

No, yes, it gets a little tricky (?) and remembering that, remembering it at the right times, etc., yes, when the day is over and tomorrow and all this, right ?? otherwise, no, it worked.

- It worked. What was it like to record this physical activity with this stick figure and press it?

Sorry, I didn't hear correctly.

- How did you like recording physical activity via this stick figure?

Yeah, I was just filling in the time there, basically, when I was working and...

- Yes and how much did you fill in then, it sounds like you had to fill in a lot then?

Yes, it was a little, it was a little every day, but okay, as you know, the time was many hours I spent doing that.

- Yes, exactly, and when did you do this?

Yes, how often did I do it? I tried to do it every day, tried to do it, but sometimes I made a few mistakes.

- You say, what do you say, was it in the evening or in the morning?

Yes, it was both.

- Okay.

One at 5 o'clock and...

- You got up at 5 in the morning ..

I get up at 4-5 every morning.

- Oh.

Well, then I might as well go out and work.

- But then, did you also register activity in the morning?

Well, not right away, maybe around 8 o'clock.

- Oh, oh, oh, oh, yes.

?? We tried to fill it in as quickly as possible, but it was a little here and there.

- Yes and how is it, do you want to tell me here and there you say, how did you experience it?

Sometimes I remembered it and sometimes I didn't, and then my wife took over and she didn't remember everything either, so we tried to keep track of each other there.

- Yes, you did that every day, right?

As far as it went, but it was probably skipped for a day.

- How did you do it then, were you able to go back then or not?

No, I didn't go back and do anything, no, no.

- No.

I don't remember the number of hours myself, if it had been a day it would have been gone the next day, like I don't remember???

- Well, what you had filled in the day before was gone, you mean?

Not gone but that exact hours, what did I do then.

- You think you don't remember, do you mean?

Exactly ???

- And this, can you describe a little more about how it was difficult?

Yes, but if you miss and fill it in and then think back a day later and so on, it's not that easy then, no???

- No, you don't remember, do you mean?

First of all, go back to that device, no one told me.

- No, I don't think it did.

No, it wasn't that easy.

- Yes, that's right, I'm wondering a bit because you had it for 12 weeks anyway..

Yes.

- .. and then it was like, I don't know if you saw it but you could set a goal for the coming week and for each week there was a summary of how much activity you had used and so you could set goals for the coming week, did you do that?

No, no, I had no idea about that.

- You didn't know that?

No, and I'm not that technically gifted, if I may say so, and I understand that kind of stuff so it won't be that much, no.

- No, so no one showed you that?

No, maybe it was, but not everything works.

- No, okay, and you didn't click on that because you were a little curious, right?

No, I'm not that curious, at least not about that, no.

- Because then it is also the case that there is also a tab on this screen, now you don't see it anymore but there was a tab called history...

I think I saw it.

- Yes, but did you do anything with it?

No, no, no.

- Nothing.

What I did was weigh myself every morning and then I filled in some statistics with hours and how I felt and yeah?? etc. what it was, probably should have and all that ? That's what I did with that device.

- But activity then you say that you, you didn't do anything like that ?

No, no, I only wrote for hours when I was active.

- Yes, for this one, you know, this when you saw it as a small 10-minute thing, you know ..

No, no ????

Wife: You filled in how long you were doing it and it was, well, 10 or 20 minute intervals.

- Exactly, didn't you?

Yes, yes, but it took a couple of hours in total.

- Yes, I understand, was it your wife who did it many times then?

Yes, we helped each other with that, yes.

- Then you can press the right number of times if it was several hours per day?

???

- What did you say?

Yes, it happened many times.

- Yes, it was. I hear that and how was it then that there was so much, was it like, what did you think about it?

Yeah, I don't know, I have to work for it and make it automatically(?)

- Could it be made better in some way, could it be improved in some way?

I know, what do you mean?

Wife: You mean if it were any other way?

- Yes?

Wife: I don't know, it wasn't hard to print that either.

It was just a matter of pressing a number of times and counting the minutes.

- No, I'm just wondering if you thought about whether there were any suggestions for improvement for us and to take action because I think you ...

??? hours had done nothing.

- What did you say?

I said hours...

Wife : ???

- But that could be a choice, you mean?

Yes, about that.

- But this history tab for going back to it, you didn't look at it at all, did you?

No, not what I, no.

- And not your wife either?

I do n't think so , no, she says.

- Did you use this activity coach in any other way?

Did I do it, no, we say.

- And then I wonder how much you used the activity coach?

Wife: Well, but what do you mean, they pressed that old man and filled in how many, how long he had worked.

- And how often did you do that?

Wife: When we arrived, that is, when we remembered as I say, usually every day.

- Mostly every day, yes, and did you think that was a lot or a little?

What do you think now?

- Yes, I mean more if it was a lot of work or if it went well?

Wife: No, it wasn't anything difficult.

No, no.

- And was that how you had planned it to go?

I don't know ..

Wife: What do you mean, we did as we were told.

- Yes, I understand. Then some questions about this, if you see any development opportunities to make this activity coach, this stick figure a little better?

Wife: Well, we probably have no thoughts about that.

It's a little difficult.

- No, but if we could imagine that someone else would use it now, perhaps who has more need for it, how could we make it better?

Yes, that's right, I can imagine making a box for hours instead of 10 minutes.

- Yes, exactly.

For 10 minutes then (rings loudly)

- Was there something you were missing?

No, I don't know, I can't say that, no.

- Was there anything that would have made you want to use it more?

No, I don't think it would have been, I don't think so ?????

- I'm almost done here. So I'm wondering if you were offered to continue having this screen with the stick figure, the activity coach, how would you feel about it?

Yeah, I don't know, I don't know if it affects me that much in the situation I'm in now with my health and all this because I'm not affected that way. I understand that there are those who suffer much, much worse than I do who need to have it in a different way than I have, I think that's how it is.

- So if you were to receive the offer now, would you turn it down?

Yes, I believe so, I believe so.

- Have you felt any difference now, have you anyway, or was there somehow something that made you use it for 12 weeks?

Yes, it's for research purposes.

- Is it for research?

Yes, that you stand up for it all.

- You volunteered for the sake of research?

Yes.

- So actually, this thing about you getting some use for it yourself, how does it feel when I ask you again?

I don't know, of course it was fun and then how many hours you were working then if you calculate, it was fun and then see but I know that roughly anyway . It might not be that many hours if there are hours at all ??? and next time I can drive harder again.

- So you still looked into this a bit with how much you were doing during the day?

Yes, I probably did that a little grandly, yes.

- And would you like to describe a little more what you felt?

Yes, it was positive, it's always useful and I've always been very much in favor of working with the body, right ?? and being able to do it and do it in a way that's positive for me.

- So it was positive to still see, because you saw when you filled in the activity coach that it was many hours?

Yes exactly. That's what's good if you can keep it going in a fairly lukewarm way , don't be too against it???

- Yes, exactly that, and I wonder if there's anything else you've reflected on regarding this activity coach?

No, no, no not really, nice guy who came here, Andreas, he was really nice. So much so that I've even offered him coffee when he comes by.

- No, but what if you don't have anything to add?

No thanks, that's probably fine.

- No, but you know what, I'll end the recording here.

Yes.
