# Supplementary material for: Exploring user experience: A qualitative analysis of the use of a physical activity support app for people with heart failure
Source: PLoS One. 2025 May 22;20(5):e0309577. doi: 10.1371/journal.pone.0309577 (PMC12097600; doi:10.1371/journal.pone.0309577)
Supplement: S1 File — English_verbatim. (ZIP) [file pone.0309577.s001.zip › English_verbatim/THJO033_eng.docx]

**THJO033**

- Then I will ask you the first question here and please tell me what physical activity means or means to you?

Mainly walks and gymnastics with Sofia on TV now that my TV is working. It has been very bad with that.

- So, yes?

Yes.

- Then let's see, can you give some other examples of what you could think of as physical activity?

Absolutely, snow shoveling, which there has been very little of this winter, but gardening, which is now starting to require more and more effort.

- Exactly, yes. How do you think about physical activity in relation to the fact that you have this diagnosis of heart failure, the symptoms it brings with it, what do you think about that?

I think physical activity is great.

- And how are you affected or limited to any extent, do you choose activities based on your symptoms as well or how does it affect physical activity in your life?

Yes, it is true that in the last 3-4 weeks I have noticed a catastrophic decline in my fitness, probably based on too much sitting during the winter months, so that even when I walk up the basement stairs, 12 steps, I feel like I walk like a really old man plus I have become very wobbly. So I can't walk completely straight but have to lean against the walls sometimes when I walk in the hallway or in a room or the bathroom or when I walk outside, so I prefer to have walking poles.

- And what made you want to volunteer and participate in this research project?

General curiosity that research is very interesting regardless of the field.

- What expectations did you have before the participation began, so to speak?

That I would get support and a kick to more regularly do those walks and yes, barbell exercises and gymnastics exercises that I have had a bit of difficulty doing for a couple of years now.

- Did you experience to what extent these expectations were met?

Hardly at all.

- Can you develop?

Yes, I have become a tired old man who doesn't have that capacity even though I know mentally that I should but I don't.

- Ok, then this is going to be a pretty broad question and then I just want to remind you again that we are now talking about the part of this screen that was about physical activity and then I would like to ask you to tell me about your experiences of using the activity coach?

It's great. It's just that the scale doesn't really work in communication with this plate all the time so I'm really sick of it when I'm standing there half-frozen in the morning and have stepped out of my night clothes and am completely naked, I can't get it to work until maybe after 5-10 minutes where I have to restart the screen maybe not just once but a couple of times but stubborn as I am, I usually succeed in the end and then the weight comes up.

- Yeah, okay.

?? I'm so pissed off.

- Yes, I can believe that, but ...

And I think it's about what mmHG we have , so if there are a lot of clouds and if it's light and beautiful outside and the weather changes, then it seems like this device turns off and then I have to press again and press again, yes and restart but now I've learned that. I can manage it just fine without having to call you or anyone else.

- And this part then, the activity coach, again and register with this plus then, how much physically active you have been, how has that affected you during the course of the experiment?

Yes, dear one, I think this little push has been positive every time I've looked at it daily.

- Would you say or how has that use affected physical activity?

Yes, on a scale of 1-10 , it has probably affected me at least a 5.

- Okay, yes, did you experience any negative, did you have any negative experiences or experiences of working with the activity coach and physical activity?

No, but I would also like to add that after the 4th and 5th Corona shots I was completely knocked out. After the 4th, I lay down for 3 days without doing anything other than getting up and peeing, drinking water and going to sleep when I woke up and not wanting to eat. After the 5th shot, when they took a break for 4-5 months, the same result was achieved, but then my knock-out lasted for 2 days and both times I had terrible pain in my shoulder area, shoulders and lower back and the strange thing is that after the last one, which was maybe 2½ months ago, this evil is still in my body and I can't get rid of it and the doctor can't really handle this problem.

- It wasn't fun. How long has it been now?

Yes, I think that since this 5th injection it must have been, I think it was in February.

- Oops.

Yes, so much so that I really suffer from it and feel bad and I scream in pain. I won't say that I scream for a long time but it's like ah ah oh it hurts when I get out of bed or straighten up when I'm lying on my back and have to turn my body sideways, I wake up at night because it hurts and I have a very hard time falling back asleep because it hurts and that goes all the way up to my biceps, shoulders, arms and down to pretty much my tailbone. It's crap, it's crap, I have so much to do in the garden.

- Yes, it was negative of course then. If we turn it around then, do you have any positive experiences from using this activity coach? You may have answered that to some extent, but I'll ask the question anyway?

Yes, the positive thing is that through your friendly treatment and instructions you have encouraged me to carry out my daily activities in a better way and with more continuity, although I must admit that recently, also because since November my big TV has not worked very well, which means that from time to time I miss the morning exercises with Sofia at 10. There are only pixels and no sound and then it jumps and then it goes out and then I have learned to use my small phone so that I can , well, I don't watch Sofia on the phone, I must admit, but I can at least watch Aktuellt when it's not working or on some other program and that's great, by the way, because then I can choose what time or when I'm done with the dishes or whatever it is and I want to sit down and watch. It's absolutely excellent, then I don't get stressed.

- No, that's it ..

Half past seven starts, yes one of the news programs, not Aktuellt but the other one, yes and then I always get stressed to go in and watch it from the beginning. Now I can come in at 5:00 and watch the whole program by watching it on my phone. I would have a TV like that so I can connect it when I want and yes it is connected to this gymnastics exercises that I could actually also manage but I sleep, God forbid, from 12 at night until about 9.30 in the morning and then I don't always have time to finish the toilet until 10 when Sofia starts but then it doesn't bother me in the same way. I am infinitely grateful that she is there and has the strength to hold me under her arms, so to speak, several times a week.

- Yes, that's good, ok. Then let's see here, to register physical activity in this activity coach, you press this stick figure, so to speak, and then you get this view with the jar where you can add or fill in these 10-minute steps about physical activity, how did you experience that procedure or that process, so to speak?

Yes, it was really nice, so if you could put more than 1 kroner in that pile, so to speak, it was very positive. It was stimulating, yes.

- Can you elaborate more on how you thought and perhaps felt about it?

Yes, it was more like a pat on the back, good boy.

- Then we'll see, let's see if this is something that you noted, every week on Monday when the previous week's activity was summarized on the screen and you were also given the opportunity to set a goal to achieve for the coming week, is it something that you recognize and if so, how did you experience it?

It was also positive, yes, setting goals is always nice and I have learned to not only set goals but also a time frame within which certain things should be fulfilled because this situation means that we humans, and I speak for myself a lot, have a tendency to postpone until tomorrow what you can do the day after tomorrow.

- Yes, it was a nice version.

Yes indeed, it has haunted me my whole life but it's actually, I don't know, what's the prost thing called ... prostatagradia?

- Prostaglandin is a hormone, but procrastinators ...

procrastination , yes, that's what it's called. I've heard the scouts talk about it in a program I listen to on P1...

- Yes, yes, I usually listen to that too.

.. a few years ago, yes, but there are several who threw it in front of them and give it a shit even though they know it should have been done...

- Yes, I think it's a human ..

.. and it's easily done.

- .. a human condition. Great, then we'll see, can you tell us more about that particular goal, whether you experienced it as positive, negative or what emotions you got, whether it was stressful or motivating, etc.?

Absolutely not stressful, but on the contrary, more motivating and therefore when you see that you have achieved a goal you are happy, while when you get a little slap on the fingers when you have not achieved your goal, slap on the fingers.

- Then we'll see, another functionality that I don't know if you used, so you get that of course, affects the answer then, but there is a history tab on this screen where you, where you have already been able to look at weight etc. over time, but there you could also look at the activity over time, is that something that you did and can you share any experiences with this?

I don't remember if I did. It's extremely rare that I go into the current and old screen, the same screen that I've had for several years, and look at these curves because they look pretty much the same.

- Yes, I understand.

Yes, there will be no major change. However, a warning came up today that I should contact the Lena Andersson health center in Flen because I have gained 2 kg in the last 3 days, which does not really match the notes I have made. I called Lena but got no answer and I know she would say, yes, but then you have to take and pick up a few more Furix .

- Yes, that's usually the case.

There was a period, I was up to 94 kg here, had slowly gained weight over a few weeks. Day by day by day the weight increased and then I thought about what she had said earlier, yes but if there is a weight gain then just take one or a couple more Furix per day and I did that for 14 days and the weight trudged so slowly downwards and then suddenly it jumped in the last 3 days which cannot be true with the device's display ability so to speak plus a small detail that yesterday or if it was the day before yesterday this schedule came up where you ask questions about ..

- Symptoms perhaps?

Sorry about?

- About symptoms, no?

No, what does it say, well how are you, how are you feeling basically , and there are 3 different questions and I answered them pretty much the same way every time so there is no change. That's why the curves are completely ?? you could say ..

- Yes but then ??

.. now this bastard's question came up today or the 3 questions came up again today after only 2 days.

- Yes, it's because if the system feels that you have gained weight quickly and you are then asked to call healthcare, it may be good and valuable for them that you have had to answer these symptom questions one more time, that's the idea.

Yeah, okay.

- So that was it, so to speak, it's supposed to work that way.

Well, then maybe I should add that I stopped taking Furix a little over a week ago when I had come down from 94 kg to about 89.6 and it stayed around 89.1-89.7 and so on for a few days and then I thought then I don't need to take Furix anymore. It's because I've been given an additional preparation for the heart by Sister Delli there at the health center and it's called Forxiga , Forxiga 10 mg ..

- Yes, that's exactly what it is ..

.. then she said that it is against.. therefore you don't need to continue taking Furix because it contains a little bit of, let's call it a propellant to empty water from your body in these Forxigas but since I still gained 94 kg, I started taking Furix and then I stopped taking it about a week ago and that's also because if I run errands outside the home then it's a bit inconvenient to have taken another diuretic tablet like this because then you kind of have to be prepared to find a toilet somewhere.

- Exactly, I understand. No, it's common, of course, but it's good that you've been given that medicine. It's new and should be prescribed to everyone with failure, really.

Yes, on 10/3 I look at the box. I took this out, so I started with it and it's 10 mg so it's 1 + 0 + 0, so one in the morning and nothing more.

- Well then we'll see here, and I'll go back to my form here and the next question I have is, we've talked here about registering physical activity, we've talked about goals, we've talked about the history tab and in light of that I would just like to ask the question, do you use the activity coach in any other way or for anything other than what we've talked about?

No, I don't know how one could use it in that case, but I drive according to the regulations.

- Absolutely fine. Then let's see here, how much would you say the activity coach used?

Every morning, yes or in the morning at about 10 am .

- Did you experience it as a lot or a little?

No, it seems completely okay and it's been this long so I don't read what these guys and gals say on the screen and sometimes a text like this comes up where you have to read a lot, a continuation of the comments so you can find out a little more about what heart failure means, etc. but I think I 've read that once or twice in the 3 years or whatever, maybe 4 years that I've had the device so I guess I'm a bit careless there. Maybe I should read more often.

- If we put it this way, as much as you actually use it, was it in line with what you had set out to do, so to speak? Was that roughly what you thought?

Yes, yes, I've just been told to weigh myself every morning.

- Exactly, and now we're going to change forums here or what should I say, focus a little more on the last questions here and maybe get a little more speculative or think, but if you were allowed to think freely here, do you see anything that you think should be developed with this particular physical activity coach to make it better?

Yes, that was a great question. How much commission do I get on it?

- Yes, that is justified.

You should have asked that question already when you decided to consult me, so to speak.

- Yes, that would have been wise perhaps.

Because it was a bit surprising and I don't have a direct answer on how to develop it, I must say.

- If we put it this way, was there anything you felt worked less well?

No, nothing more than that damn device not feeling so good depending on what high and low pressure we have because that's when it shifts and turns itself off, so to speak. Yes, it doesn't turn itself off, but it doesn't show the weight and that's when I have to keep fiddling with that button at the bottom right and reset it and restart the screen sometimes.

- Was there anything related to recording physical activity that you were missing?

Would you be kind enough to address that question?

- In the context when you registered physical activity with this plus sign and so on, was there something that you were missing in that process, something that would have made you experience greater value from the registration?

No.

- I take some notes on the side too.

It was so long ago that I've almost forgotten it.

- If we put it this way, is there anything that you feel would have made you want to use it more?

Yes, there was a long pause for thought here.

- Yes, it's going well.

No, you can delete this as you go along. No, I think it's very appropriate. It would be possible to be forced to use the scale morning and evening, if that makes any difference, because it has happened that I have weighed myself without it being registered because it was registered in the morning or in the afternoon and then I may have eaten a big meal. I'm very careless with my food. I can eat lunch at 1, 2, 3, 4, 5 ...

- Oops.

I get so incredibly tired afterwards that I like to go and sleep after dinner but then I thought when I weighed myself the next morning, well what did I eat last night because I weigh more today. Well that's exactly what it was and I keep careful notes in my diary here too about what I eat for breakfast and lunch and it's usually just one cooked meal a day and very rarely coffee and a coffee bun or something, well once a day at most but then in the evening I want to fill up with a little bit of food and then like the other evening it can be chicken for lunch and potatoes and vegetables boiled and raw and then in the evening I felt no I wasn't enough so then I took some sausage and fried it up and potatoes and a fried egg and it felt pleasant but at the same time I know that tomorrow morning I will weigh a little more even though I have a fantastic function when it comes to emptying my bowels. I don't really have time to get up and sit on the toilet, so sometime around 9:30 I empty my bowels quite a lot. However, I can't, yes I also pee 2 times a night as a rule and in the morning or early afternoon when I get up, but when I sit there I can empty urine quite well, but it takes and hear and amaze between 20 and 30 minutes before I feel that I have also emptied my bladder enough so that there will be no post-blockage (?) and it's a bit scary because this has been going on for, well, almost a year and it has only gotten worse and worse. At the beginning I could sit for 10 minutes, then it has increased to a quarter of an hour, today it is 20 minutes to half an hour and then I feel that now I am ready to get up from here but now I have finished urinating. There is not much working time left. You sleep 10-12 hours a day and then you sit on the cup for half an hour now and then.

- Have you sought help for this?

Sorry?

- Have you sought treatment for it?

No, I haven't done that. It's been many years since they operated on my prostate and I know it was a bit awkward afterwards and they sent me a really long, I shouldn't say tube but it looked like a tube and it was a bit rounded, pointed at one end and you had to moisten it and insert it into the urethra and all the way up there or down into the bladder to empty everything but that was so awkward and nasty and I had to keep doing it because it wasn't something I had time to get used to either and then I also had to learn that you shouldn't empty your bladder completely because there should always be a little slop so to speak left at the bottom of the bladder but this isn't so good and I don't want to report this. I'm a bit embarrassed to do it, I can tell you that. I have a lot of trust in you as you understand and then it's also the case that I don't want them to go in and start poking around in my penis again. I also have n't had a woman in 10-11 years and I can't get an erection, I can try to masturbate once in a while and it's a bit hard. I can have a semi-orgasm but it's not much more either so it's almost not worth it for what you get you have to pay for it like the auntie on the train once to me and that didn't apply then but it costs about half an hour, an hour of sleep after I've masturbated and that's not fun either but the sex drive is there. I dream of beautiful angels sometimes, I can wake up and feel a desire that remains after the dream but it doesn't seem to have any results. I can look, I've joined Facebook God forbid and there they sometimes show women with big tits and I like that. Who doesn't do that, it's been there since motherhood when you had to suck on your mother's tits which were quite big in parentheses and yes it is, it's a part of life that I miss, I guess. If you have 5 spokes in a wheel and you remove one spoke, it will limp a bit, right ?

- Yes, it was a bit lateral there, but if I jump back in here to the list of questions, if you, if you were offered the opportunity to continue using this activity coach, so that it would be part of the screen that you have today, how would you view it?

Yes, it is part of my daily life so I have no objection to continuing and the registration is done via the device even though I am zealous and as you know I like tables and stuff so I enter this into my agenda every day. I can see here that on Monday I weighed 88.4, today I weighed 91.2 just as an example and there I also write down what temperature we have and there I also have a separate list that I have prepared with date and time what the temperature is and what I weigh in the morning and if I have dreamed or if I have had a loose stomach in the morning and when I get up and pee and how I usually have my bowel movements in the morning very regularly. Today there was nothing in itself but as a rule it works perfectly. I have had tables for several months if you are interested.

- Then I have the last formal question here before we interrupt the formal part of the conversation and it is also one of these, now you will probably accuse me of wanting to know the question prematurely here and I will probably have to blush with shame but do you have anything else you want to reflect on, highlight or tell about this with physical activity and participation in the project and so on?

Yes, it is in that case and it comes naturally here now that you have a personal contact and not just go into a computer and look at the screen. Well, this is how it looks today and this is how it looks, you can go back and look at what it looked like the last 3-4 weeks for example, but this personal support is very important, I think.

- Do you think it would be in the form of a messaging function or a call or something like that ..

Yes, a conversation. Just as a kind of follow-up, maybe once a month or every other month anyway. I've often thought about you here lately, not just as a researcher so to speak, but as a person too. I wonder how you and your little ones, etc. are doing back home.

- We're going through everyday life. Yes, but listen, that was the last question, so if there's nothing else you want to add, I feel ready to stop recording.

Yes, the interview is over there.

- Then I will leave a formal thank you.
