# Supplementary material for: Exploring user experience: A qualitative analysis of the use of a physical activity support app for people with heart failure
Source: PLoS One. 2025 May 22;20(5):e0309577. doi: 10.1371/journal.pone.0309577 (PMC12097600; doi:10.1371/journal.pone.0309577)
Supplement: S1 File — English_verbatim. (ZIP) [file pone.0309577.s001.zip › English_verbatim/ULKY097_eng.docx]

**ULKY097**

- Then I thought a little bit, just a little bit to refresh my memory, you had or do you still have that screen with the activity meter at home?

Yes.

- Do you have that?

Yes.

- And what do you use it for now?

Yes, I mostly use it to weigh myself and then I use it, I feel like I have the same amount of activities that I do almost every week so there is no variation.

- No, I understand. Because that's what Andreas calls the stick figure, that's what I want to ask you a little bit about today.

Yes, and I've been bad at it.

- But I'll ask a few questions anyway, so you can tell me what you can, I think.

Yes.

- I thought I'd start with just a little general question, can you tell me what physical activity means to you?

It's that I go to my husband's nursing home. I walk a lot? And then I move around here in the apartment. It's physical activity. I used to go to aqua gym and sit-ups, but I don't anymore. For me, it's physical activity.

- That's physical activity for you. Anything else you can tell me about your thoughts on physical activity?

Now I don't understand what you said.

- Well, I wonder if there's anything else you can think of that is physical activity for you?

No.

- No, but you go to the nursing home and then you go home to the apartment?

Yes, and then I go to town, like now, for example, I'm going to walk for half an hour and meet two friends and we're going to go out for lunch.

- Yes, how nice.

And then I don't really know where we're walking.

- No, I understand that, so you're out on the town sometimes too and go there?

Yes, at least when the weather is nice.

- Yes and that's it today?

Yes.

- How wonderful. I know that you have this diagnosis of heart failure and so I wonder if you have anything you think about physical activity in relation to having this heart failure diagnosis?

I've been sad thinking about it.

- You've been sad and thinking about it, can you just describe it a little more what you mean by that?

Yes, I think that when I think about death, it can come and I can't die yet for my children. My daughter has died and my grandchildren are very, I am important to them.

- So when I ask the question about exercise and heart failure, it's like, you jump straight to your death, right?

Yes, I think about death.

- Yes, I understand how you've been dealing with it, it's very tragic for you, I hear.

Yes, but I try to push it away so that at least when I meet my grandchildren, I try to think about something else.

- What do you mean then, think about other things, repress what do you mean?

Yes, I think about life..

- On life ..

.. on a ?

- And now that I ask about heart failure and activity, can you just or do you not want to answer that at all?

I don't know, I'm trying to push it away, I should say.

- Yes, I understand.

It's only when I have really hard time breathing and when my chest feels tight that I actually think something is wrong.

- And how is it, what do you think about physical activity then, are you somewhat limited in your activity?

If?

- Are you limited?

Yes, yes, when I get one like that, yes, not exactly painful, but annoying.

- Busy, hm, and what do you do then?

Yes, when it feels really heavy, I go to bed. I lie on the bed and try to breathe deeply and think about something else.

- Yes, and does this happen when you are out and about or when does this happen?

It can come at any time and then I know that my blood pressure is also like that, jumping up and down. It doesn't get any better to keep measuring it.

- , but if you ... because when you're out and about in town and with your friends now, is that something you think about or are you limited in any way, how do you think about that?

No, I just think it'll be fun.

- So you don't have any symptoms of your heart failure when you go down to town?

Most of the time not. It might come at some point.

- Sometime.

That I get it. There is pressure in my chest but then I walk slower.

- Yes, then you go slowly, yes. Then the next question comes, I wonder what made you want to participate in this research project with the activity coach?

I thought but it's good if someone comes up with something and wants to work on it. That's just what I thought and he was very nice to talk to. At that time we had been to the police and were warned to let someone in and there were probably 100 of us and 2 police officers and there had been so many robberies here in town so I was probably very bored. I said that we had been warned to let someone in but he was so nice to talk to so I forgot about it.

- And you talked about something, you said it was good for something but you're welcome to elaborate a little bit on this that you wanted to participate. Then you said something about and then I'm just wondering if you want to elaborate a little bit?

Yes, that something would happen, that something would be invented and done. I don't know, I don't know what I was thinking. I just thought that it would be great if someone noticed it.

- And that, what do you mean by that?

I mean, then you would have to come up with something that you could do.

- And do something what?

Yes that would be better or prevent, yes ..

- What did you say?

Or prevent it from happening, from getting worse. Yeah, I don't know, I can't express myself.

- And did you have any expectations before participating in this study? You said it a little bit recently, but ..

I didn't have any special expectations, but I thought it was fun that someone was interested in it.

- And interested in?

Yes, from the heart.

- No, you're talking about expecting ... you said something about getting better, what were your thoughts on that?

Yes, if you could, you would think it would be worth it to get more active , that it would help you, but it's true that some days after a walk it's really nice and other days I have to go to bed when I get home.

- Were these expectations met?

I can't say exactly.

- No.

I didn't have any special expectations when it's like this that you can become too old like I am. I notice what I did 5 years ago and what I do now that there is a difference in energy and everything.

- Then I'm wondering if you can tell me about your experiences using this activity coach, the stick figure?

Yes, sometimes I'm very diligent and remember it and press it and then a week goes by and I don't remember it.

- How often did you remember it then?

Yes, sometimes often and then a whole week can go by and I don't remember it. I remember it when I weigh myself. I think that when I'm panting more or when my chest feels heavy, that's when I remember it. Then I weigh myself and I think I've probably gained a couple of kilos. Not that I care about it but I think I have, that I've accumulated fluid but then yeah I don't know what happens it goes away and when I feel good I actually forget about it.

- So this activity coach, the stick figure, did you press it when you weighed yourself or what should I say?

Yes, after that I've printed it and then I've been really good and then I've remembered when I come from, for example, this nursing home where my husband is. Then I remember that I'm going to print now but I'm very careless that I often forget and ?

- Yes, how does this affect you when you use this stick figure, has it affected you?

" I would use it more anyway, but I don't think so, it depends on whether other things are important.

- What do you mean then?

Yes, everything that happens to my grandchildren affects me.

- If we go back to the activity coach, has it affected your physical activity in any way?

Did you say psychic?

- So physical, so your training you could say or your activity, has this activity coach affected you in any way?

Yes, some, that moment or what should I say, was a bit grand.

- Yes and how?

I think of it when I see it in the kitchen and I remember what I did today.

- What do you remember then?

Yes, for example, I've been to town and then I think to myself that it's over a km.

- And then you tick that, then you have used the activity coach and ticked that you have walked then?

Yes, but I don't do it every day and not every week.

- Yes, I understand, I just wanted to ask, this question too, has it kind of affected your physical activity in any way?

I can't really answer that because it's like this that I, I feel really good some days and then I feel worse and then it's both mentally and physically.

- Yes, I understand.

So that I feel like this that just, well, if I've had a headache and a heavy chest, then I just think about whether I might have a stroke now or something like that, and it's probably that I'm afraid of dying deep down.

- Okay. Did you experience any negative effects from using this activity coach, the stick figure?

No, it's not a negative thing. Sometimes I get really happy when I see it.

- You'll be happy?

Yes, I think I need to toughen up now.

- Yeah, and you're thinking about sharpening your skills?

And come on and do my lines.

- Your streaks yes. Were there any pos .. yes you say that but did you experience any positive experiences from using this activity coach more?

No, it's just that I think that when I've done it I think what have I done today but then I don't know, it's really hard to know I think for example when I had this pedometer on me so every time I felt really bad and could almost only lie in bed and I thought poor whoever is going to read this. There were no steps and counting.

- That's not really what it's about, you shouldn't feel this demand that you have to do something, you just want to capture reality.

Yes, but you know, I think it's like, I want that kind of reality that would confirm that I've moved.

- I see.

That I haven't just been lying in bed, but it's much easier to go out now than when it's snowing and slippery. Then I have a walker.

- I understand. How was it, how did you experience it and register in this stick figure, how was it?

Yes, it took a long time before I figured out how to do it and remember because my memory has also gotten worse, but I was happy every time I found the old man.

- What did you say now?

I was happy when I found the old man.

- So you were happy when you pushed this guy?

Yes.

- So then I wonder, it used to be that every week your activity was summarized on the screen and you could set a goal for the coming week, did you use it and how did you experience it if so?

No, I didn't use it, I never understood it.

- You haven't understood?

No.

- No, and I'm wondering, there was a tab where you could look at the history going back in time, did you use it at all or anything?

No, I came across those people who had various problems.

- Yes, but there was one on this computer screen itself, so you could press a history, a tab where you could see how you were, what activity you had done during the weeks, but did you use it at all?

No.

- Not at all?

No, I don't think so.

- Was there any other way you used the activity coach other than ...

Even though I made those lines?

- Yes, anything else you used it for?

No, I read about people there.

- Sorry?

I read about different people, they were women and men.

- Okay, so you were inside reading?

Yes.

- Then the question arises, how much did you use this activity coach?

I can't answer that for you. Should I say hours or weeks?

- Yes, you can try and, you don't have to say hours.

I can't answer how much.

- Was it every week or was it, not every day, I understand?

Yes, it was every week anyway.

- That was it, so several times a week?

Sometimes several times and sometimes nothing.

- I understand, did you think it was a lot or a little?

It was a bit much if I think about it, but I should have thought better.

- No and was it roughly in line with how you had intended to use it?

I can't answer that. I didn't have any special thoughts like that.

- No, is there anything else you see that we could develop with this activity coach to make it better?

Yes, I don't know what to say. I said that I've benefited the most from being able to weigh myself, from being able to think that yes, that it hasn't gotten worse.

- Yes, exactly, and you mean by weight?

Yes, because I actually know one two three, you can weigh 2 kg more and then I think it's great that I know that and now I've taken some Furosemide (?). I can pee out 2 liters.

- It's fantastic to have it that way and be able to control it yourself.

Yes.

- But if we go back to the activity coach, was there anything that worked less well?

Not anything, I had no expectations that it would see that it was not good. It was, yes, of course it would have more teaching, maybe I would have to tighten up a bit.

- More teaching?

Hmm.

- And then comes the next question, what were you missing?

Yes, what was I missing, I think it was that I should have read this history that I haven't found.

- If there was something that would have made you want to use it more, can you think of something?

I think so. It's kind of like how my grandchildren have taught me how to use a cell phone, I could, I didn't understand anything and now I can do it, I understand a little.

- But this question, what was it that would like, what was it that had made you want to use it more, was it that you would, what were you thinking there?

No, but that was the one I would like to try more, I couldn't find that one ..

- The history?

Yes.

- Okay, you didn't find it?

No.

- Exactly, no, then maybe you would have used it more, you mean?

Yes.

- If you were now offered to continue using this activity coach, how would you feel about it?

I should probably use it a little more because now I notice that I can't do anything (?)..

- Can you explain why you say that?

Yes, but I think I would get more benefit from it then maybe.

- Yes and what do you mean, can you describe it a little more?

Yes, I would, if there was something I could do that would help me and understand better.

- And understand better?

Yes.

- And then comes the last question, is there anything else you have reflected on or highlighted that you would like to share with us before we end?

No, I can't think of anything that comes to mind. But it was good to talk to you because then I know there's more to it.

- Yes, it does, yes of course you still have it so you can look at it now.

Yes.

- And you can set, you could set goals too. Now let's see here, and then, because you didn't look at it, you didn't look at it every week, the sum that you had typed in was summed up. Did you see that you could see then the following week, that you could set new goals?

What do you mean by goal?

- In this activity computer, in the computer, you could sum up the activities every week and then you could ..

And what other activities are there than moving around?

- No, what you had pressed, what you have moved every week, you see every week how much, then you get, the first week you got a sum and then you could yourself but you hadn't used that part, then you could have gone in and set goals for the following week, did you do that?

No.

- No, but then you know, then you still have it if you want, you can try it now.

Yes, now I'm going to Eskilstuna.

- Now I think that unless you have anything more to add, we will end the interview.

Yes.

- Then I'll turn off the recording here.
