# Supplementary material for: Exploring user experience: A qualitative analysis of the use of a physical activity support app for people with heart failure
Source: PLoS One. 2025 May 22;20(5):e0309577. doi: 10.1371/journal.pone.0309577 (PMC12097600; doi:10.1371/journal.pone.0309577)
Supplement: Attachment — Submitted filename: Response to Reviewers.docx [file pone.0309577.s003.docx]

PONE-D-24-34455

Exploring User Experience: A Qualitative Analysis of the use of a Physical Activity Support App for People with Heart Failure

PLOS ONE

Dear Dr. Blomqvist,

Thank you for submitting your manuscript to PLOS ONE. After careful consideration, we feel that it has merit but does not fully meet PLOS ONE’s publication criteria as it currently stands. Therefore, we invite you to submit a revised version of the manuscript that addresses the points raised during the review process.

Please submit your revised manuscript by Nov 29 2024 11:59PM. If you will need more time than this to complete your revisions, please reply to this message or contact the journal office at <a href="mailto:plosone@plos.org">plosone@plos.org. When you're ready to submit your revision, log on to https://www.editorialmanager.com/pone/ and select the **'Submissions Needing Revision'** folder to locate your manuscript file.

Please include the following items when submitting your revised manuscript:

- **A rebuttal letter that responds to each point** raised by the academic editor and reviewer(s). You should upload this letter **as a separate file** labeled 'Response to Reviewers'.
- **A marked-up copy of your manuscript that highlights changes** made to the original version. You should upload this as a separate file labeled 'Revised Manuscript with Track Changes'.
- An **unmarked version of your revised paper without tracked changes**. You should upload this as a separate file labeled 'Manuscript'.

If you would like to make changes to your financial disclosure, please include your updated statement in your cover letter. Guidelines for resubmitting your figure files are available below the reviewer comments at the end of this letter.

If applicable, we recommend that you deposit your laboratory protocols in protocols.io to enhance the reproducibility of your results. Protocols.io assigns your protocol its own identifier (DOI) so that it can be cited independently in the future. For instructions see: <https://journals.plos.org/plosone/s/submission-guidelines#loc-laboratory-protocols>. Additionally, PLOS ONE offers an option for publishing peer-reviewed Lab Protocol articles, which describe protocols hosted on protocols.io. Read more information on sharing protocols at <https://plos.org/protocols?utm_medium=editorial-email&utm_source=authorletters&utm_campaign=protocols>.

We look forward to receiving your revised manuscript.

Kind regards,

Najmul Hasan, PhD
Academic Editor
PLOS ONE

Journal requirements:

When submitting your revision, we need you to address these additional requirements.

1. Please ensure that your manuscript meets PLOS ONE's style requirements, including those for file naming. The PLOS ONE style templates can be found at

https://journals.plos.org/plosone/s/file?id=wjVg/PLOSOne_formatting_sample_main_body.pdf and

<https://journals.plos.org/plosone/s/file?id=ba62/PLOSOne_formatting_sample_title_authors_affiliations.pdf>

2. Thank you for stating the following in the Competing Interests section: [I have read the journal's policy and the authors of this manuscript have the following competing interests:

The author Andreas Blomqvist, PhD-student at Linköping University, is an employee and shareholder of the company that developed the app and Optilogg (CareLigo AB, Sweden).].

Please confirm that this does not alter your adherence to all PLOS ONE policies on sharing data and materials, by including the following statement: "This does not alter our adherence to PLOS ONE policies on sharing data and materials.” (as detailed online in our guide for authors http://journals.plos.org/plosone/s/competing-interests). If there are restrictions on sharing of data and/or materials, please state these. Please note that we cannot proceed with consideration of your article until this information has been declared. Please include your updated Competing Interests statement in your cover letter; we will change the online submission form on your behalf.

3. In the online submission form, you indicated that [All data is available through request to the corresponding author.]. All PLOS journals now require all data underlying the findings described in their manuscript to be freely available to other researchers, either 1. In a public repository, 2. Within the manuscript itself, or 3. Uploaded as supplementary information. This policy applies to all data except where public deposition would breach compliance with the protocol approved by your research ethics board. If your data cannot be made publicly available for ethical or legal reasons (e.g., public availability would compromise patient privacy), please explain your reasons on resubmission and your exemption request will be escalated for approval.

Additional Editor Comments (if provided):


[Note: HTML markup is below. Please do not edit.]

Reviewers' comments:

Reviewer's Responses to Questions

**Comments to the Author**

1. Is the manuscript technically sound, and do the data support the conclusions?

The manuscript must describe a technically sound piece of scientific research with data that supports the conclusions. Experiments must have been conducted rigorously, with appropriate controls, replication, and sample sizes. The conclusions must be drawn appropriately based on the data presented.

Reviewer #1: Partly

Reviewer #2: Partly

2. Has the statistical analysis been performed appropriately and rigorously?

Reviewer #1: N/A

Reviewer #2: No

3. Have the authors made all data underlying the findings in their manuscript fully available?

The [PLOS Data policy](http://www.plosone.org/static/policies.action#sharing) requires authors to make all data underlying the findings described in their manuscript fully available without restriction, with rare exception (please refer to the Data Availability Statement in the manuscript PDF file). The data should be provided as part of the manuscript or its supporting information, or deposited to a public repository. For example, in addition to summary statistics, the data points behind means, medians and variance measures should be available. If there are restrictions on publicly sharing data—e.g. participant privacy or use of data from a third party—those must be specified.

Reviewer #1: Yes

Reviewer #2: No

4. Is the manuscript presented in an intelligible fashion and written in standard English?

PLOS ONE does not copyedit accepted manuscripts, so the language in submitted articles must be clear, correct, and unambiguous. Any typographical or grammatical errors should be corrected at revision, so please note any specific errors here.

Reviewer #1: Yes

Reviewer #2: Yes

5. Review Comments to the Author

Please use the space provided to explain your answers to the questions above. You may also include additional comments for the author, including concerns about dual publication, research ethics, or publication ethics. (Please upload your review as an attachment if it exceeds 20,000 characters)

Reviewer #1: Hello,
Thanks for your submission on this manuscript titled "Exploring User Experience: A Qualitative Analysis of the use of a Physical Activity Support App for People with Heart Failure" to this journal. I have following suggestions and seek clarifications -
1. The sample size of the study is very small (10) and this study should have been just a proof of concept while many such studies exist for various other apps . can you please explain why the sample size was so small ? Please mention the same as limitation of the study.

Under “Ethical approval and population” we describe the reason for the small sample size. A pilot-RCT was performed using the intervention (“the activity coach”). All study participants from that pilot-RCT who were randomized to the intervention arm were approached to participate in this study, and all who conceded were included.

Under “Study design and limitations” we write that “it might have added value to have had a larger study population”. We have however, as suggested by the reviewer, also elaborated on sample size in the context of reflexive thematic analysis in this Limitations-section.

2. Were the questions ever validated in past ?

The questions in the interview guide were created based on other published interview guides from similar contexts (Anderson et al. Mobile health apps to facilitate self-care. PloS one. 2016; and Schueller et al. Understanding people’s use of and perspectives on mood-tracking apps. JMIR mental health. 2021)

3. What was the median/mean duration of each question answered and time from which the interview was done post use of app ?

This information has been added under “Data collection”.

4. Please elaborate more on the app technicalities
Thanks

This has been elaborated on under “The mHealth tool”.

Reviewer #2: Thank you for the opportunity to review this manuscript, which addresses an important and timely issue regarding the use of mobile health (mHealth) technologies to promote physical activity in patients with heart failure. While this is an important topic with potential public health benefits, there are major shortcomings that need to be addressed, including study design, lack of technological innovation, methodological limitations, insufficient depth of analysis, and overall rigor of the study. Below, I outline these concerns in detail.

Major Concerns:
1. This study presents interesting results, but is limited by its small sample size (n=10). The statistical methods used are not sufficient to support the study conclusions, which weakens the generalizability and reliability of the results. More rigorous statistical techniques and a larger dataset are necessary to validate the results. Furthermore, while thematic analysis is appropriate for qualitative research, this manuscript lacks depth in discussing the practical implications of the results in a broader clinical context.

Thank you. In this paper we used a qualitative method, aimed to describe the experiences in a qualitative way, and not focus on statistics. We used reflexive thematic analysis, where statistical considerations are not appropriate but rather the narratives of the users are important.

With regards to the sample size, we have elaborated the discussion on the topic under “Study design and limitations”.

2. Additionally, the app under study lacks technological innovation. While the app is a step in the right direction, the current version does not offer any unique features compared to existing fitness tracking apps and therefore does not demonstrate significant innovation. There is no explanation to the connection between this app and heart failure.

The Activity Coach specifically addresses so called incidental or non-exercise physical activity (NEPA), which is different than exercise (e.g. used in “fitness tracking apps”). This NEPA affects prognosis in the heart failure population. While the body of science on exercise interventions for the heart failure population is extensive, there is to our knowledge no published science on mHealth interventions specifically targeting NEPA in the HF community. As such, this is a rather unique intervention.

The authors thus maintain that this is a poorly studied field, and one that is very important as the heart failure population need support in NEPA according to leading bodies on cardiovascular prevention and rehabilitation.

3. There are contradictions within the results section. For instance, while Line 190-191 indicate that users experienced challenges in tracking physical activities, Line 199 suggests that many users found the app easy to navigate. This contradiction raises concerns about the data's reliability and the manuscript's internal consistency, and these contradictions and their implications for real-world use are not sufficiently addressed.

We understand this confusion. The challenge was in “defining physical activity”, not the concrete tracking of it in the app. We have clarified this under “Theme 1 – Cultivating awareness of physical activity engagement”.

4. The study focuses on short-term use (12 weeks), but long-term behavior change is crucial for managing heart failure. A longer follow-up period or more discussion on long-term engagement is needed.

The authors agree that long-term data would be valuable, and we have added this in the discussion.

5. The manuscript lacks an in-depth usability testing framework, which is essential for evaluating the app's effectiveness, especially given the target audience of elderly users. Additionally, the study lacks more in-depth analysis of how the app is targeted to attract user behaviors through UX terminology and methodologies. The overall user experience is spoken at too general of a level and fails to tap into describing key users’ tasks that are critical in describing the entire experience.

The aim of the study was to “describe users’ experiences”, not to assess usability nor to describe underlying theory. Both usability and aspects of how behaviours are targeted has been studied and published previously (Blomqvist, 2024, BMC Medical Informatics and Decision Making, Usability and feasibility analysis of an mHealth-tool).

Minor:
1. There is a potential conflict of interest because one of the authors is an employee and shareholder of the company that developed the app being evaluated. This relationship was disclosed, but there is insufficient information about how the study mitigated bias through independent assessment or objective safeguards regarding the study design, data collection, or interpretation of results.

Thank you for pointing this out to us and the opportunity to clarify this. We have added some information as to how this potential bias was mitigated, under “Data collection”.

2. Figure 1 should be improved in quality, and Table 2 would benefit from additional data points, such as age, gender, and symptom classification, to provide more context to the analysis.

We have updated the figure with higher resolution.

Table 2 contains age and gender and NYHA classification is a classification of the levels of heart failure related symptoms.

3. There is inconsistency regarding the target audience In regard to age group, the introduction emphasizes the elderly, but Line 356 & 358 refer to "younger users," causing confusion about the app's intended user base. The study initially indicated that the focus for the app as well is for those with HF, but in the findings indicated that users additionally had other conditions, which could have also influenced the user’s behaviors in relation to using the app.

Thank you, we have corrected this. Regarding other conditions, we listed the baseline demographics in Table 2, so the reader can have a better understanding of the health status of the studied population.

4. The study also shows concerns about the transparency of the data, not only because of the weak quantitative analysis, but also because of the questionable availability of the raw data according to the journal's policy.

A strictly qualitative methodology was used to ensure trustworthiness. We will make sure to clarify that all data in the form of verbatim transcripts are available, according to the journal’s policy.

5. The manuscript contains several grammatical errors and unclear sections. For example, vague terms such as “symptoms” need to be clarified, and more clarity is needed in the introduction, particularly in linking the purpose of the study to the gaps in existing research

Thank you, we have gone through the manuscript and have tried to improve on grammar and language clarity.

6. Line 61: Unclear how “portable” address to line 54-55 “comorbidities, … and transportation”

Thank you, this has been clarified.

7. Line 95-96: “if the user…. About 75-100 words” ◊ Does this really matter?

Thank you for this comment. While developing the app, we found published literature on mHealth adoption and usability, which indicate that finding a balance between ease of use (i.e. not too “cluttered” appearance) and easily accessed information is important. This was mentioned to clarify how that was achieved, although we concede it might be superfluous.

8. Line 182: “Cultivating awareness…” ◊ Does this refer to awareness of an individual and their physical activity.

Yes. We have clarified in “Theme 1 – Cultivating awareness of physical activity engagement”, where we describe what that theme means.

9. Line 190-191: “users did experience some challenges in defining and effectively tracking their physical activities during the daily use” is contradiction to Line 199 “many users found the app easy to navigate and use”

We clarified this. Please see the answer to “Major concerns #3” for a more elaborate response.

10. Line 268-273: Who is the target audience for the app?

The mHealth-tool designed for supporting physical activity in people with HF, which implicitly means that the majority of users will likely be elderly. While we under “Discussion” discuss whether it might be more suitable for people who are physically inactive, the current manuscript did not employ such criteria.

11. Line 356 & 358: “younger users” & “ independent of age” ◊ In introduction mentions usability of elderly

We believe that the most likely user of the app will be elderly, and as a consequence usability for elderly users was believed to be important.

In conclusion, while the manuscript addresses an important area of mHealth applications for individuals with heart failure, the manuscript requires significant revisions in methodology, innovation, and clarity to meet the standards for publication. I encourage the authors to address these concerns and resubmit, as I believe this study has the potential to make a meaningful contribution to the field.

We thank the reviewer for constructive comments. We maintain that the methodology being strictly qualitative is sound, and not methodologically flawed by not being quantitative in nature, as suggested. We also thank the reviewer for the encouraging concluding remark.

6. PLOS authors have the option to publish the peer review history of their article ([what does this mean?](https://journals.plos.org/plosone/s/editorial-and-peer-review-process#loc-peer-review-history)). If published, this will include your full peer review and any attached files.


If you choose “no”, your identity will remain anonymous but your review may still be made public.


**Do you want your identity to be public for this peer review?** For information about this choice, including consent withdrawal, please see our [Privacy Policy](https://www.plos.org/privacy-policy).

Reviewer #1: **Yes:** DR KAMAL H SHARMA

Reviewer #2: No
